# Supplementary material for: Identifying Causal Genotype–Phenotype Relationships for Population‐Sampled Parent–Child Trios
Source: Genet Epidemiol. 2026 Jan 11;50(1):e70027. doi: 10.1002/gepi.70027 (PMC12793723; doi:10.1002/gepi.70027)
Supplement: Supplementary file 1 — Supplementary Information [file GEPI-50-0-s001.pdf]

Supporting Information for
Identifying causal genotype-phenotype relationships for population-sampled parent-child trios

Yushi Tang*, Irineo Cabreros†, and John D. Storey*,[1]

*Lewis-Sigler Institute for Integrative Genomics and †Program in Applied and Computational Mathematics, Princeton University, NJ 08544, USA

[1]Corresponding author: jstorey@princeton.edu

Contents

A Theory **1**

A.1 Proof of Lemma 1 1

A.2 Proof of Lemma 2 1

A.3 Proof of Lemma 3 2

A.4 Proof of Lemma 4 Part A 4

A.5 Proof of Lemma 4 Part B 6

A.6 Proof of Lemma 5 9

A.7 Proof of Lemma 7 10

A.8 Proof of Lemma 8 12

A.9 Proof of Lemma 9 15

A.10 Proof of Lemma 10 17

A.11 Proof of Lemma 11 17

A.12 Proof of Lemma 12 18

A.13 Proof of Lemma 13 19

A.14 Relationship to the direct-indirect trio model 20

B Simulations **21**

B.1 Simulating trio genotypes 21

B.2 Simulating quantitative trait 21

B.3 Permutation test 22

B.4 Simulating dichotomous trait 22

B.5 Simulating genetic linkage 22

B.6 Simulating confounding effects 23

C Supplementary Figures **24**

References **32**

A Theory

A.1 Proof of Lemma 1

Under the trait model in Equation (1),

$$\begin{aligned}\text{ACE}(A^m \to Y) + \text{ACE}(A^p \to Y) &= \mathbb{E}[Y^m(1)] - \mathbb{E}[Y^m(0)] + \mathbb{E}[Y^p(1)] - \mathbb{E}[Y^p(0)] \\ &= (\alpha_1 - \alpha_0)\mathbb{P}(A^p = 0) + (\alpha_2 - \alpha_1)\mathbb{P}(A^p = 1) \\ &\quad + (\alpha_1 - \alpha_0)\mathbb{P}(A^m = 0) + (\alpha_2 - \alpha_1)\mathbb{P}(A^m = 1).\end{aligned}$$

Assume $0 < \mathbb{P}(A^m = 1) < 1$ and $0 < \mathbb{P}(A^p = 1) < 1$. Under Assumption 1, either $\alpha_0 \leq \alpha_1 \leq \alpha_2$ or $\alpha_0 \geq \alpha_1 \geq \alpha_2$. Then $\text{ACE}(A^m \to Y) + \text{ACE}(A^p \to Y) = 0$ if and only if $\alpha_0 \neq \alpha_1$ or $\alpha_1 \neq \alpha_2$, which means $\text{ACE}(G \to Y) \neq 0$ by Definition 2.

A.2 Proof of Lemma 2

Here we show $\Big(Y^m(0), Y^m(1)\Big) \perp\!\!\!\perp A^m | Z^m = 1$. The proof for $\Big(Y^p(0), Y^p(1)\Big) \perp\!\!\!\perp A^p | Z^p = 1$ is the same. We first show that $A^p \perp\!\!\!\perp A^m | Z^m = 1$ through several calculations.

$$\begin{aligned}&\mathbb{C}(A^m, A^p | Z^m = 1) \\ &= \mathbb{E}[A^m A^p | Z^m = 1] - \mathbb{E}[A^m | Z^m = 1]\mathbb{E}[A^p | Z^m = 1] \\ &= \mathbb{P}(A^m = 1, A^p = 1 | Z^m = 1) - \mathbb{P}(A^m = 1 | Z^m = 1)\mathbb{P}(A^p = 1 | Z^m = 1) \\ &= \mathbb{P}(A^m = 1 | Z^m = 1)\mathbb{P}(A^p = 1 | A^m = 1, Z^m = 1) - \frac{1}{2}\mathbb{P}(A^p = 1 | Z^m = 1) \\ &= \frac{1}{2}\mathbb{P}(A^p = 1 | A^m = 1, Z^m = 1) - \frac{1}{2}\mathbb{P}(A^p = 1 | Z^m = 1). \qquad \text{(A2.a)}\end{aligned}$$

By the Law of Total Covariance,

$$\begin{aligned}&\mathbb{C}(A^m, A^p | Z^m = 1) \\ &= \mathbb{E}\Big[\mathbb{C}(A^m, A^p | Z^m = 1, Z^p = 1)\Big| Z^m = 1\Big] \\ &\qquad + \mathbb{C}\Big(\mathbb{E}[A^m | Z^m = 1, Z^p = 1], \mathbb{E}[A^p | Z^m = 1, Z^p = 1]\Big| Z^m = 1\Big) \\ &= \mathbb{E}\Big[\mathbb{E}[A^m A^p | Z^m = 1, Z^p = 1] - \mathbb{E}[A^m | Z^m = 1, Z^p = 1]\mathbb{E}[A^p | Z^m = 1, Z^p = 1]\Big| Z^m = 1\Big] \\ &\qquad + \underbrace{\mathbb{C}\Big(\frac{1}{2}, \frac{1}{2}\Big| Z^m = 1\Big)}_{=0}\end{aligned}$$

$$= \mathbb{E}\Big[\frac{1}{4} - \frac{1}{2}\cdot\frac{1}{2}\Big| Z^m = 1\Big]$$
$$= 0. \tag{A2.b}$$

Since Equation (A2.a) and Equation (A2.b) are equal, this implies $\mathbb{P}(A^p = 1|A^m = 1, Z^m = 1) - \mathbb{P}(A^p = 1|Z^m = 1) = 0$. Similarly, $\mathbb{P}(A^p = b|A^m = a, Z^m = 1) = \mathbb{P}(A^p = b|Z^m = 1)$ for $a, b \in \{0, 1\}$. So

$$A^m \perp\!\!\!\perp A^p | Z^m = 1. \tag{A2.c}$$

By Assumption 2,

$$\mathbb{P}(A^m = a|Z^m = 1) = \frac{1}{2} = \mathbb{P}(A^m = a|Z^m = 1, \gamma), \quad a \in \{0, 1\},. \tag{A2.d}$$

Under the trait model in Equation (1), $Y^m(0)$ and $Y^m(1)$ are functions of A^p and γ. By Equation (A2.c) and Equation (A2.d), we have shown that A^p and γ are independent of A^m conditional on $Z^m = 1$. Thus, $\big(Y^m(0), Y^m(1)\big)|Z^m = 1$ and $A^m|Z^m = 1$ are independent.

A.3 Proof of Lemma 3

$$\mathbb{E}[d^{\text{nc}}_{\text{TMT}}|N] = \mathbb{E}\left[\frac{2}{N}\left(\sum_{j=1}^{J} Y_j W_{1j} - \sum_{j=1}^{J} Y_j W_{0j}\right)\middle| N\right]$$
$$= \frac{2}{N}\sum_{j\in\mathcal{J}} \mathbb{E}[W_{1j}Y_j|N] - \frac{2}{N}\sum_{j\in\mathcal{J}} \mathbb{E}[W_{0j}Y_j|N]$$
$$= \frac{2}{N} J\mathbb{E}[W_1 Y|N] - \frac{2}{N} J\mathbb{E}[W_0 Y|N]$$
$$= \frac{2J}{N}\mathbb{E}\Big[\mathcal{I}(A^m = 1, Z^m = 1)Y + \mathcal{I}(A^p = 1, Z^p = 1)Y\Big|N\Big]$$
$$- \frac{2J}{N}\mathbb{E}\Big[\mathcal{I}(A^m = 0, Z^m = 1)Y + \mathcal{I}(A^p = 0, Z^p = 1)Y\Big|N\Big]$$
$$= \frac{2J}{N}\mathbb{E}\Big[\mathcal{I}(A^m = 1, Z^m = 1)\big(Y^m(0)\mathcal{I}(A^m = 0) + Y^m(1)\mathcal{I}(A^m = 1)\big)\Big|N\Big]$$
$$+ \frac{2J}{N}\mathbb{E}\Big[\mathcal{I}(A^p = 1, Z^p = 1)\big(Y^p(0)\mathcal{I}(A^p = 0) + Y^p(1)\mathcal{I}(A^p = 1)\big)\Big|N\Big]$$
$$- \frac{2J}{N}\mathbb{E}\Big[\mathcal{I}(A^m = 0, Z^m = 1)\big(Y^m(0)\mathcal{I}(A^m = 0) + Y^m(1)\mathcal{I}(A^m = 1)\big)\Big|N\Big]$$
$$- \frac{2J}{N}\mathbb{E}\Big[\mathcal{I}(A^p = 0, Z^p = 1)\big(Y^p(0)\mathcal{I}(A^p = 0) + Y^p(1)\mathcal{I}(A^p = 1)\big)\Big|N\Big]$$
$$= \frac{2J}{N}\mathbb{E}\Big[\mathcal{I}(A^m = 1, Z^m = 1)Y^m(1)\Big|N\Big]$$

$$
\begin{aligned}
&+ \frac{2J}{N}\mathbb{E}\Big[\mathcal{I}(A^p = 1, Z^p = 1)Y^p(1)\Big|N\Big] \\
&- \frac{2J}{N}\mathbb{E}\Big[\mathcal{I}(A^m = 0, Z^m = 1)Y^m(0)\Big|N\Big] \\
&- \frac{2J}{N}\mathbb{E}\Big[\mathcal{I}(A^p = 0, Z^p = 1)Y^p(0)\Big|N\Big] \\
= &\frac{2J}{N}\mathbb{E}\Big[\mathbb{E}\big[\mathcal{I}(A^m = 1)\mathcal{I}(Z^m = 1)Y^m(1)\big|Z^m = 1, N\big]\Big|N\Big] \\
&+ \frac{2J}{N}\mathbb{E}\Big[\mathbb{E}\big[\mathcal{I}(A^p = 1)\mathcal{I}(Z^p = 1)Y^p(1)\big|Z^p = 1, N\big]\Big|N\Big] \\
&- \frac{2J}{N}\mathbb{E}\Big[\mathbb{E}\big[\mathcal{I}(A^m = 0)\mathcal{I}(Z^m = 1)Y^m(0)\big|Z^m = 1, N\big]\Big|N\Big] \\
&- \frac{2J}{N}\mathbb{E}\Big[\mathbb{E}\big[\mathcal{I}(A^p = 0)\mathcal{I}(Z^p = 1)Y^p(0)\big|Z^p = 1, N\big]\Big|N\Big] \\
= &\frac{2J}{N}\mathbb{E}\Big[\mathcal{I}(Z^m = 1)\mathbb{E}\big[\mathcal{I}(A^m = 1)Y^m(1)\big|Z^m = 1, N\big]\Big|N\Big] \\
&+ \frac{2J}{N}\mathbb{E}\Big[\mathcal{I}(Z^p = 1)\mathbb{E}\big[\mathcal{I}(A^p = 1)Y^p(1)\big|Z^p = 1, N\big]\Big|N\Big] \\
&- \frac{2J}{N}\mathbb{E}\Big[\mathcal{I}(Z^m = 1)\mathbb{E}\big[\mathcal{I}(A^m = 0)Y^m(0)\big|Z^m = 1, N\big]\Big|N\Big] \\
&- \frac{2J}{N}\mathbb{E}\Big[\mathcal{I}(Z^p = 1)\mathbb{E}\big[\mathcal{I}(A^p = 0)Y^p(0)\big|Z^p = 1, N\big]\Big|N\Big] \qquad \text{(A3.a)} \\
= &\frac{2J}{N}\mathbb{P}\big(Z^m = 1\big|N\big)\mathbb{E}\big[\mathcal{I}(A^m = 1)Y^m(1)\big|Z^m = 1, N\big] \\
&+ \frac{2J}{N}\mathbb{P}\big(Z^p = 1\big|N\big)\mathbb{E}\big[\mathcal{I}(A^p = 1)Y^p(1)\big|Z^p = 1, N\big] \\
&- \frac{2J}{N}\mathbb{P}\big(Z^m = 1\big|N\big)\mathbb{E}\big[\mathcal{I}(A^m = 0)Y^m(0)\big|Z^m = 1, N\big] \\
&- \frac{2J}{N}\mathbb{P}\big(Z^p = 1\big|N\big)\mathbb{E}\big[\mathcal{I}(A^p = 0)Y^p(0)\big|Z^p = 1, N\big] \qquad \text{(A3.b)} \\
= &\frac{2J}{N}\mathbb{P}\big(Z^m = 1\big|N\big)\mathbb{E}\big[\mathcal{I}(A^m = 1)\big|Z^m = 1, N\big]\mathbb{E}\big[Y^m(1)\big|Z^m = 1, N\big] \\
&+ \frac{2J}{N}\mathbb{P}\big(Z^p = 1\big|N\big)\mathbb{E}\big[\mathcal{I}(A^p = 1)\big|Z^p = 1, N\big]\mathbb{E}\big[Y^p(1)\big|Z^p = 1, N\big] \\
&- \frac{2J}{N}\mathbb{P}\big(Z^m = 1\big|N\big)\mathbb{E}\big[\mathcal{I}(A^m = 0)\big|Z^m = 1, N\big]\mathbb{E}\big[Y^m(0)\big|Z^m = 1, N\big] \\
&- \frac{2J}{N}\mathbb{P}\big(Z^p = 1\big|N\big)\mathbb{E}\big[\mathcal{I}(A^p = 0)\big|Z^p = 1, N\big]\mathbb{E}\big[Y^p(0)\big|Z^p = 1, N\big] \qquad \text{(A3.c)} \\
= &\frac{2J}{N}\mathbb{P}\big(Z^m = 1\big|N\big)\mathbb{P}\big(A^m = 1\big|Z^m = 1, N\big)\mathbb{E}\big[Y^m(1)\big|Z^m = 1, N\big] \\
&+ \frac{2J}{N}\mathbb{P}\big(Z^p = 1\big|N\big)\mathbb{P}\big(A^p = 1\big|Z^p = 1, N\big)\mathbb{E}\big[Y^p(1)\big|Z^p = 1, N\big] \\
&- \frac{2J}{N}\mathbb{P}\big(Z^m = 1\big|N\big)\mathbb{P}\big(A^m = 0\big|Z^m = 1, N\big)\mathbb{E}\big[Y^m(0)\big|Z^m = 1, N\big] \\
&- \frac{2J}{N}\mathbb{P}\big(Z^p = 1\big|N\big)\mathbb{P}\big(A^p = 0\big|Z^p = 1, N\big)\mathbb{E}\big[Y^p(0)\big|Z^p = 1, N\big] \qquad \text{(A3.d)}
\end{aligned}
$$

$$
\begin{aligned}
&= \frac{2J}{N}\cdot\frac{N}{2J}\cdot\frac{1}{2}\mathbb{E}\big[Y^m(1)\big|Z^m=1,N\big] + \frac{2J}{N}\cdot\frac{N}{2J}\cdot\frac{1}{2}\mathbb{E}\big[Y^p(1)\big|Z^p=1,N\big]\\
&\quad - \frac{2J}{N}\cdot\frac{N}{2J}\cdot\frac{1}{2}\mathbb{E}\big[Y^m(0)\big|Z^m=1,N\big] - \frac{2J}{N}\cdot\frac{N}{2J}\cdot\frac{1}{2}\mathbb{E}\big[Y^p(0)\big|Z^p=1,N\big] \qquad \text{(A3.e)}\\
&= \frac{1}{2}\mathbb{E}\big[Y^m(1)-Y^m(0)\big|Z^m=1,N\big] + \frac{1}{2}\mathbb{E}\big[Y^p(1)-Y^p(0)\big|Z^p=1,N\big]\\
&= \delta_{\mathrm{TMT}}.
\end{aligned}
$$

Equation (A3.a) is equal to Equation (A3.b) by the Law of Total Probability. Equation (A3.b) is equal to Equation (A3.c) by Lemma 2 in that $Y(0), Y(1) \perp\!\!\!\perp A \,\big|\, Z = 1$. Equation (A3.d) is equal to Equation (A3.e) since $\mathbb{P}(Z_j^m = 1|N) = \mathbb{P}(Z_j^p = 1|N) = N/(2J)$, which follows because all parents are exchangeable and there are $2J$ parents.

A.4 Proof of Lemma 4 Part A

$$
\begin{aligned}
\mathbb{E}[\hat{\mu}_c|N] &= \mathbb{E}\left[\frac{1}{N}\left(\sum_{j\in\mathcal{J}} Y_j W_{1j} + \sum_{j\in\mathcal{J}} Y_j W_{0j}\right)\middle| N\right]\\
&= \frac{1}{N}\sum_{j\in\mathcal{J}}\mathbb{E}[W_{0j}Y_j|N] + \frac{1}{N}\sum_{j\in\mathcal{J}}\mathbb{E}[W_{1j}Y_j|N]\\
&= \frac{1}{N}J\mathbb{E}[W_0Y|N] + \frac{1}{N}J\mathbb{E}[W_1Y|N]\\
&= \frac{J}{N}\mathbb{E}\Big[\mathcal{I}(A^m=0,Z^m=1)Y + \mathcal{I}(A^p=0,Z^p=1)Y\Big|N\Big]\\
&\quad + \frac{J}{N}\mathbb{E}\Big[\mathcal{I}(A^m=1,Z^m=1)Y + \mathcal{I}(A^p=1,Z^p=1)Y\Big|N\Big]\\
&= \frac{J}{N}\mathbb{E}\Big[\mathcal{I}(A^m=0,Z^m=1)\big(Y^m(0)\mathcal{I}(A^m=0) + Y^m(1)\mathcal{I}(A^m=1)\big)\Big|N\Big]\\
&\quad + \frac{J}{N}\mathbb{E}\Big[\mathcal{I}(A^p=0,Z^p=1)\big(Y^p(0)\mathcal{I}(A^p=0) + Y^p(1)\mathcal{I}(A^p=1)\big)\Big|N\Big]\\
&\quad + \frac{J}{N}\mathbb{E}\Big[\mathcal{I}(A^m=1,Z^m=1)\big(Y^m(0)\mathcal{I}(A^m=0) + Y^m(1)\mathcal{I}(A^m=1)\big)\Big|N\Big]\\
&\quad + \frac{J}{N}\mathbb{E}\Big[\mathcal{I}(A^p=1,Z^p=1)\big(Y^p(0)\mathcal{I}(A^p=0) + Y^p(1)\mathcal{I}(A^p=1)\big)\Big|N\Big]\\
&= \frac{J}{N}\mathbb{E}\Big[\mathcal{I}(A^m=0,Z^m=1)Y^m(0)\Big|N\Big]\\
&\quad + \frac{J}{N}\mathbb{E}\Big[\mathcal{I}(A^p=0,Z^p=1)Y^p(0)\Big|N\Big]\\
&\quad + \frac{J}{N}\mathbb{E}\Big[\mathcal{I}(A^m=1,Z^m=1)Y^m(1)\Big|N\Big]\\
&\quad + \frac{J}{N}\mathbb{E}\Big[\mathcal{I}(A^p=1,Z^p=1)Y^p(1)\Big|N\Big]
\end{aligned}
$$

$$
\begin{aligned}
&= \frac{J}{N}\mathbb{E}\Big[\mathbb{E}\big[\mathcal{I}(A^m = 0)\mathcal{I}(Z^m = 1)Y^m(0)\big|Z^m = 1, N\big]\Big|N\Big] \\
&\quad + \frac{J}{N}\mathbb{E}\Big[\mathbb{E}\big[\mathcal{I}(A^p = 0)\mathcal{I}(Z^p = 1)Y^p(0)\big|Z^p = 1, N\big]\Big|N\Big] \\
&\quad + \frac{J}{N}\mathbb{E}\Big[\mathbb{E}\big[\mathcal{I}(A^m = 1)\mathcal{I}(Z^m = 1)Y^m(1)\big|Z^m = 1, N\big]\Big|N\Big] \\
&\quad + \frac{J}{N}\mathbb{E}\Big[\mathbb{E}\big[\mathcal{I}(A^p = 1)\mathcal{I}(Z^p = 1)Y^p(1)\big|Z^p = 1, N\big]\Big|N\Big] \\
&= \frac{J}{N}\mathbb{E}\Big[\mathcal{I}(Z^m = 1)\mathbb{E}\big[\mathcal{I}(A^m = 0)Y^m(0)\big|Z^m = 1, N\big]\Big|N\Big] \\
&\quad + \frac{J}{N}\mathbb{E}\Big[\mathcal{I}(Z^p = 1)\mathbb{E}\big[\mathcal{I}(A^p = 0)Y^p(0)\big|Z^p = 1, N\big]\Big|N\Big] \\
&\quad + \frac{J}{N}\mathbb{E}\Big[\mathcal{I}(Z^m = 1)\mathbb{E}\big[\mathcal{I}(A^m = 1)Y^m(1)\big|Z^m = 1, N\big]\Big|N\Big] \\
&\quad + \frac{J}{N}\mathbb{E}\Big[\mathcal{I}(Z^p = 1)\mathbb{E}\big[\mathcal{I}(A^p = 1)Y^p(1)\big|Z^p = 1, N\big]\Big|N\Big] && \text{(A4.a)} \\
&= \frac{J}{N}\mathbb{P}\big(Z^m = 1\big|N\big)\mathbb{E}\big[\mathcal{I}(A^m = 0)Y^m(0)\big|Z^m = 1, N\big] \\
&\quad + \frac{J}{N}\mathbb{P}\big(Z^p = 1\big|N\big)\mathbb{E}\big[\mathcal{I}(A^p = 0)Y^p(0)\big|Z^p = 1, N\big] \\
&\quad + \frac{J}{N}\mathbb{P}\big(Z^m = 1\big|N\big)\mathbb{E}\big[\mathcal{I}(A^m = 1)Y^m(1)\big|Z^m = 1, N\big] \\
&\quad + \frac{J}{N}\mathbb{P}\big(Z^p = 1\big|N\big)\mathbb{E}\big[\mathcal{I}(A^p = 1)Y^p(1)\big|Z^p = 1, N\big] && \text{(A4.b)} \\
&= \frac{J}{N}\mathbb{P}\big(Z^m = 1\big|N\big)\mathbb{E}\big[\mathcal{I}(A^m = 0)\big|Z^m = 1, N\big]\mathbb{E}\big[Y^m(0)\big|Z^m = 1, N\big] \\
&\quad + \frac{J}{N}\mathbb{P}\big(Z^p = 1\big|N\big)\mathbb{E}\big[\mathcal{I}(A^p = 0)\big|Z^p = 1, N\big]\mathbb{E}\big[Y^p(0)\big|Z^p = 1, N\big] \\
&\quad + \frac{J}{N}\mathbb{P}\big(Z^m = 1\big|N\big)\mathbb{E}\big[\mathcal{I}(A^m = 1)\big|Z^m = 1, N\big]\mathbb{E}\big[Y^m(1)\big|Z^m = 1, N\big] \\
&\quad + \frac{J}{N}\mathbb{P}\big(Z^p = 1\big|N\big)\mathbb{E}\big[\mathcal{I}(A^p = 1)\big|Z^p = 1, N\big]\mathbb{E}\big[Y^p(1)\big|Z^p = 1, N\big] && \text{(A4.c)} \\
&= \frac{J}{N}\mathbb{P}\big(Z^m = 1\big|N\big)\mathbb{P}\big(A^m = 0\big|Z^m = 1, N\big)\mathbb{E}\big[Y^m(0)\big|Z^m = 1, N\big] \\
&\quad + \frac{J}{N}\mathbb{P}\big(Z^p = 1\big|N\big)\mathbb{P}\big(A^p = 0\big|Z^p = 1, N\big)\mathbb{E}\big[Y^p(0)\big|Z^p = 1, N\big] \\
&\quad + \frac{J}{N}\mathbb{P}\big(Z^m = 1\big|N\big)\mathbb{P}\big(A^m = 1\big|Z^m = 1, N\big)\mathbb{E}\big[Y^m(1)\big|Z^m = 1, N\big] \\
&\quad + \frac{J}{N}\mathbb{P}\big(Z^p = 1\big|N\big)\mathbb{P}\big(A^p = 1\big|Z^p = 1, N\big)\mathbb{E}\big[Y^p(1)\big|Z^p = 1, N\big] && \text{(A4.d)} \\
&= \frac{J}{N}\cdot\frac{N}{2J}\cdot\frac{1}{2}\mathbb{E}\big[Y^m(0)\big|Z^m = 1, N\big] + \frac{J}{N}\cdot\frac{N}{2J}\cdot\frac{1}{2}\mathbb{E}\big[Y^p(0)\big|Z^p = 1, N\big] \\
&\quad + \frac{J}{N}\cdot\frac{N}{2J}\cdot\frac{1}{2}\mathbb{E}\big[Y^m(1)\big|Z^m = 1, N\big] + \frac{J}{N}\cdot\frac{N}{2J}\cdot\frac{1}{2}\mathbb{E}\big[Y^p(1)\big|Z^p = 1, N\big] && \text{(A4.e)} \\
&= \frac{1}{2}\Big\{\frac{1}{2}\big(\mathbb{E}\big[Y^m(0)\big|Z^m = 1, N\big] + \mathbb{E}\big[Y^p(0)\big|Z^p = 1, N\big]\big)
\end{aligned}
$$

$$+\frac{1}{2}\big(\mathbb{E}\big[Y^m(1)\big|Z^m=1,N\big]+\mathbb{E}\big[Y^p(1)\big|Z^p=1,N\big]\big)\Big\}$$
$$=\frac{1}{2}\Big\{\mu_0+\mu_1\Big\}=\mu_c$$

This completes the proof for Lemma 4. Equation (A4.a) is equal to Equation (A4.b) by the Law of Total Probability. Equation (A4.b) is equal to Equation (A4.c) by Lemma 2 in that $Y(0),Y(1)\perp\!\!\!\perp A\,\big|\,Z=1$. Equation (A4.d) is equal to Equation (A4.e) because $\mathbb{P}(Z_j^m=1|N)=\mathbb{P}(Z_j^p=1|N)=N/(2J)$, , which follows because all parents are exchangeable and there are $2J$ parents.

A.5 Proof of Lemma 4 Part B

$$\begin{aligned}
&\frac{2J}{N}\mathbb{E}[(W_{1j}-W_{0j})\mu_c|N]\\
&=\frac{2J}{N}\mu_c\mathbb{E}\Big[\mathcal{I}(A_j^m=1,Z_j^m=1)-\mathcal{I}(A_j^m=0,Z_j^m=1)\\
&\qquad+\mathcal{I}(A_j^p=1,Z_j^p=1)-\mathcal{I}(A_j^p=0,Z_j^p=1)\Big|N\Big]\\
&=\frac{2J}{N}\mu_c\mathbb{E}\Big[A_j^m\mathcal{I}(Z_j^m=1)-(1-A_j^m)\mathcal{I}(Z_j^m=1)\\
&\qquad+A_j^p\mathcal{I}(Z_j^p=1)-(1-A_j^p)\mathcal{I}(Z_j^p=1)\Big|N\Big]\\
&=\frac{2J}{N}\mu_c\mathbb{E}\Big[(2A_j^m-1)\mathcal{I}(Z_j^m=1)+(2A_j^p-1)\mathcal{I}(Z_j^p=1)\Big|N\Big]\\
&=\frac{2J}{N}\mu_c\Big(\mathbb{E}[2A_j^m-1|Z_j^m=1,Z^p\neq1,N]\mathbb{P}(Z_j^m=1,Z_j^p\neq1|N)\\
&\qquad\quad+\mathbb{E}[2A_j^p-1|Z_j^m\neq1,Z_j^p=1,N]\mathbb{P}(Z_j^m\neq1,Z_j^p=1|N)\\
&\qquad\quad+\mathbb{E}[2A_j^m+2A_j^p-2|Z_j^m=1,Z_j^p=1,N]\mathbb{P}(Z_j^m=1,Z_j^p=1|N)\Big)\\
&=\frac{2J}{N}\mu_c\Big(0\cdot\mathbb{P}(Z_j^m=1,Z_j^p\neq1|N)+0\cdot\mathbb{P}(Z_j^m\neq1,Z_j^p=1|N)\\
&\qquad\quad+0\cdot\mathbb{P}(Z_j^m=1,Z_j^p=1|N)\Big)\\
&=0
\end{aligned}\tag{A5.a}$$

To show $\frac{2J}{N}\mathbb{E}[(W_{1j}-W_{0j})\hat{\mu}_c|N]=0$, first define

$$N_0=\sum_{k\in\mathcal{J}}\mathcal{I}(A_k^m+A_k^p=0,Z_k^m=1)+\mathcal{I}(A_k^m+A_k^p=0,Z_k^p=1),$$
$$N_1=\sum_{k\in\mathcal{J}}\mathcal{I}(A_k^m+A_k^p=1,Z_k^m=1)+\mathcal{I}(A_k^m+A_k^p=1,Z_k^p=1),$$

$$N_2 = \sum_{k\in\mathcal{J}} \mathcal{I}(A_k^m + A_k^p = 2, Z_k^m = 1) + \mathcal{I}(A_k^m + A_k^p = 2, Z_k^p = 1).$$

Notice that $N_0 + N_1 + N_2 = N$. Recall that

$$\begin{aligned} W_{1j} &= \mathcal{I}(A_j^m = 1, Z_j^m = 1) + \mathcal{I}(A_j^p = 1, Z_j^p = 1), \\ W_{0j} &= \mathcal{I}(A_j^m = 0, Z_j^m = 1) + \mathcal{I}(A_j^p = 0, Z_j^p = 1). \end{aligned}$$

Then

$$\begin{aligned} &\frac{2J}{N}\mathbb{E}[(W_{1j} - W_{0j})\hat{\mu}_c | N] \\ =& \frac{2J}{N}\mathbb{E}\Big[(W_{1j} - W_{0j})\frac{1}{N}\sum_{k\in\mathcal{J}}(W_{0k} + W_{1k})Y_k\Big|N\Big] \\ =& \frac{2J}{N^2}\mathbb{E}\Big[(W_{1j} - W_{0j})\sum_{k\in\mathcal{J}}(W_{0k} + W_{1k})(Y_k - \gamma_k)\Big|N\Big] \qquad \text{(A5.b)} \\ &\quad + \frac{2J}{N^2}\mathbb{E}\Big[(W_{1j} - W_{0j})\sum_{k\in\mathcal{J}}(W_{0k} + W_{1k})\gamma_k\Big|N\Big]. \qquad \text{(A5.c)} \end{aligned}$$

For Equation (A5.b), it equals

$$\begin{aligned} &\frac{2J}{N^2}\mathbb{E}\Big[(W_{1j} - W_{0j})\sum_{k\in\mathcal{J}}\big(\mathcal{I}(Z_k^m = 1) + \mathcal{I}(Z_k^p = 1)\big)(Y_k - \gamma_k)\Big|N\Big] \\ =& \frac{2J}{N^2}\mathbb{E}\Bigg[(W_{1j} - W_{0j})\sum_{k\in\mathcal{J}}\begin{pmatrix} \alpha_0\mathcal{I}(A_k^m + A_k^p = 0)\big(\mathcal{I}(Z_k^m = 1) + \mathcal{I}(Z_k^p = 1)\big) \\ +\alpha_1\mathcal{I}(A_k^m + A_k^p = 1)\big(\mathcal{I}(Z_k^m = 1) + \mathcal{I}(Z_k^p = 1)\big) \\ +\alpha_2\mathcal{I}(A_k^m + A_k^p = 2)\big(\mathcal{I}(Z_k^m = 1) + \mathcal{I}(Z_k^p = 1)\big) \end{pmatrix}\Bigg|N\Bigg] \\ =& \frac{2J}{N^2}\mathbb{E}\Big[(W_{1j} - W_{0j})(\alpha_0 N_0 + \alpha_1 N_1 + \alpha_2 N_2)\Big|N\Big] \\ =& \frac{2J}{N^2}\mathbb{E}\Big[\mathbb{E}\big[(W_{1j} - W_{0j})(\alpha_0 N_0 + \alpha_1 N_1 + \alpha_2 N_2)\big|N_0, N_1, N_2\big]\Big|N\Big] \\ =& \frac{2J}{N^2}\mathbb{E}\Big[\mathbb{E}\big[W_{1j} - W_{0j}|N_0, N_1, N_2\big] \cdot (\alpha_0 N_0 + \alpha_1 N_1 + \alpha_2 N_2)\Big|N\Big]. \end{aligned}$$

Note that

$$\begin{aligned} &\mathbb{E}\Big[W_{1j} - W_{0j}\Big|N_0, N_1, N_2\Big] \\ =& \mathbb{E}\Big[\mathcal{I}(A_j^m = 1, Z_j^m = 1) + \mathcal{I}(A_j^p = 1, Z_j^p = 1) \\ &\quad - \mathcal{I}(A_j^m = 0, Z_j^m = 1) - \mathcal{I}(A_j^p = 0, Z_j^p = 1)\Big|N_0, N_1, N_2\Big] \end{aligned}$$

$$
\begin{aligned}
&= \mathbb{E}\Big[A_j^m \mathcal{I}(Z_j^m = 1) + A_j^p \mathcal{I}(Z_j^p = 1) \\
&\qquad - (1 - A_j^m)\mathcal{I}(Z_j^m = 1) - (1 - A_j^p)\mathcal{I}(Z_j^p = 1)\Big| N_0, N_1, N_2\Big] \\
&= \mathbb{E}\Big[(2A_j^m - 1)\mathcal{I}(Z_j^m = 1) + (2A_j^p - 1)\mathcal{I}(Z_j^p = 1)\Big| N_0, N_1, N_2\Big] \\
&= \mathbb{E}[2A_j^m - 1 | Z_j^m = 1, Z_j^p \neq 1, N_0, N_1, N_2]\mathbb{P}(Z_j^m = 1, Z_j^p \neq 1 | N_0, N_1, N_2) \\
&\qquad + \mathbb{E}[2A_j^p - 1 | Z_j^m \neq 1, Z_j^p = 1, N_0, N_1, N_2]\mathbb{P}(Z_j^m \neq 1, Z_j^p = 1 | N_0, N_1, N_2) \\
&\qquad + \mathbb{E}[2A_j^m + 2A_j^p - 2 | Z_j^m = 1, Z_j^p = 1, N_0, N_1, N_2]\mathbb{P}(Z_j^m = 1, Z_j^p = 1 | N_0, N_1, N_2) \\
&= 0 \cdot \mathbb{P}(Z_j^m = 1, Z_j^p \neq 1 | N_0, N_1, N_2) \\
&\qquad + 0 \cdot \mathbb{P}(Z_j^m \neq 1, Z_j^p = 1 | N_0, N_1, N_2) \\
&\qquad + 0 \cdot \mathbb{P}(Z_j^m = 1, Z_j^p = 1 | N_0, N_1, N_2) \\
&= 0,
\end{aligned}
$$

and thus,

$$
\text{Equation (A5.b)} = \frac{2J}{N^2}\mathbb{E}\Big[0 \cdot (\alpha_0 N_0 + \alpha_1 N_1 + \alpha_2 N_2)\Big| N\Big] = 0.
$$

For Equation (A5.c), it equals

$$
\begin{aligned}
&\frac{2J}{N^2}\mathbb{E}\Big[(W_{1j} - W_{0j})\sum_{k \in \mathcal{J}}(W_{0k} + W_{1k})\gamma_k\Big| N\Big] \\
&= \frac{2J}{N^2}\sum_{k \in \mathcal{J}}\mathbb{E}\Big[(W_{1j} - W_{0j})(W_{0k} + W_{1k})\gamma_k\Big| N\Big] \\
&= \frac{2J}{N^2}\sum_{k \in \mathcal{J}}\mathbb{E}\Big[\big(W_{1j} - W_{0j}\big)\big(\mathcal{I}(Z_k^m = 1) + \mathcal{I}(Z_k^p = 1)\big)\gamma_k\Big| N\Big] \\
&= \frac{2J}{N^2}\sum_{k \in \mathcal{J}}\mathbb{E}\Big[(W_{1j} - W_{0j})\mathcal{I}(Z_k^m = 1)\gamma_k\Big| N\Big] && \text{(A5.d)} \\
&\qquad + \frac{2J}{N^2}\sum_{k \in \mathcal{J}}\mathbb{E}\Big[(W_{1j} - W_{0j})\mathcal{I}(Z_k^p = 1)\gamma_k\Big| N\Big]. && \text{(A5.e)}
\end{aligned}
$$

For Equation (A5.d), notice that

$$
\begin{aligned}
&\mathbb{E}\Big[(W_{1j} - W_{0j})\mathcal{I}(Z_k^m = 1)\gamma_k\Big| N\Big] \\
&= \mathbb{E}\Big[\big((2A_j^m - 1)\mathcal{I}(Z_j^m = 1) + (2A_j^p - 1)\mathcal{I}(Z_j^p = 1)\big)\mathcal{I}(Z_k^m = 1)\gamma_k\Big| N\Big] \\
&= \mathbb{E}\Big[(2A_j^m - 1)\mathcal{I}(Z_j^m = 1)\mathcal{I}(Z_k^m = 1)\gamma_k\Big| N\Big]
\end{aligned}
$$

$$
\begin{aligned}
&\quad + \mathbb{E}\Big[(2A_j^p - 1)\mathcal{I}(Z_j^p = 1)\mathcal{I}(Z_k^m = 1)\gamma_k \Big| N\Big] \\
&= \mathbb{E}\Big[(2A_j^m - 1)\gamma_k \Big| Z_j^m = 1, Z_k^m = 1, N\Big]\mathbb{P}\Big(Z_j^m = 1, Z_k^m = 1 \Big| N\Big) \\
&\quad + \mathbb{E}\Big[(2A_j^p - 1)\gamma_k \Big| Z_j^p = 1, Z_k^m = 1, N\Big]\mathbb{P}\Big(Z_j^p = 1, Z_k^m = 1 \Big| N\Big).
\end{aligned} \tag{A5.f}
$$

By the Law of Segregation,

$$
\begin{aligned}
\mathbb{P}(A_j^m = 1|Z_j^m = 1, Z_k^m = 1, N, \gamma_k) &= \frac{1}{2} = \mathbb{P}(A_j^m = 1|Z_j^m = 1, Z_k^m = 1, N), \\
\mathbb{P}(A_j^m = 0|Z_j^m = 1, Z_k^m = 1, N, \gamma_k) &= \frac{1}{2} = \mathbb{P}(A_j^m = 0|Z_j^m = 1, Z_k^m = 1, N), \\
\mathbb{P}(A_j^p = 1|Z_j^p = 1, Z_k^m = 1, N, \gamma_k) &= \frac{1}{2} = \mathbb{P}(A_j^p = 1|Z_j^p = 1, Z_k^m = 1, N), \\
\mathbb{P}(A_j^p = 0|Z_j^p = 1, Z_k^m = 1, N, \gamma_k) &= \frac{1}{2} = \mathbb{P}(A_j^p = 0|Z_j^p = 1, Z_k^m = 1, N).
\end{aligned}
$$

Then Equation (A5.f) equals

$$
\begin{aligned}
&\mathbb{E}[2A_j^m - 1|Z_j^m = 1, Z_k^m = 1, N] \cdot \mathbb{E}[\gamma_k|Z_j^m = 1, Z_k^m = 1, N] \cdot \mathbb{P}(Z_j^m = 1, Z_k^m = 1|N) \\
&\quad + \mathbb{E}[2A_j^p - 1|Z_j^m = 1, Z_k^m = 1, N] \cdot \mathbb{E}[\gamma_k|Z_j^p = 1, Z_k^m = 1, N] \cdot \mathbb{P}(Z_j^p = 1, Z_k^m = 1|N) \\
&= 0 \cdot \mathbb{E}[\gamma_k|Z_j^m = 1, Z_k^m = 1, N] \cdot \mathbb{P}(Z_j^m = 1, Z_k^m = 1|N) \\
&\quad + 0 \cdot \mathbb{E}[\gamma_k|Z_j^p = 1, Z_k^m = 1, N] \cdot \mathbb{P}(Z_j^p = 1, Z_k^m = 1|N) \\
&= 0,
\end{aligned}
$$

and thus Equation (A5.d) = 0. Similarly, Equation (A5.e) = 0. Then Equation (A5.c) = Equation (A5.d) + Equation (A5.e) = 0. Therefore, Equation (6) in Lemma 4 holds in that

$$
\frac{2J}{N}\mathbb{E}[(W_{1j} - W_{0j})\hat{\mu}_c|N] = \text{Equation (A5.b)} + \text{Equation (A5.c)} = 0.
$$

A.6 Proof of Lemma 5

$$
\begin{aligned}
\mathbb{E}[d_{\mathrm{TMT}}^{\mathrm{mc}}|N] - \mathbb{E}[d_{\mathrm{TMT}}^{\mathrm{pc}}|N] &= \mathbb{E}\Big[\frac{2}{N}\sum_{j \in \mathcal{J}}(W_{1j} - W_{0j})Y_j \Big| N\Big] \\
&\quad - \mathbb{E}\Big[\frac{2}{N}\sum_{j \in \mathcal{J}}(W_{1j} - W_{0j})(Y_j - \mu_c)\Big| N\Big] \\
&= \mathbb{E}\Big[\frac{2}{N}\sum_{j \in \mathcal{J}}(W_{1j} - W_{0j})\mu_c \Big| N\Big]
\end{aligned}
$$

$$
\begin{aligned}
&= 0 \qquad \text{(by Equation (5) in Lemma 4)} \\
\mathbb{E}[d^{\mathrm{nc}}_{\mathrm{TMT}}|N] - \mathbb{E}[d_{\mathrm{TMT}}|N] &= \mathbb{E}\Big[\frac{2}{N}\sum_{j\in\mathcal{J}}(W_{1j} - W_{0j})Y_j\Big|N\Big] \\
&\quad - \mathbb{E}\Big[\frac{2}{N}\sum_{j\in\mathcal{J}}(W_{1j} - W_{0j})(Y_j - \hat{\mu}_c)\Big|N\Big] \\
&= \mathbb{E}\Big[\frac{2}{N}\sum_{j\in\mathcal{J}}(W_{1j} - W_{0j})\hat{\mu}_c\Big|N\Big] \\
&= 0 \qquad \text{(by Equation (6) in Lemma 4)}
\end{aligned}
$$

Thus, $\mathbb{E}\left[d_{\mathrm{TMT}}|N\right] = \mathbb{E}\left[d^{\mathrm{pc}}_{\mathrm{TMT}}|N\right] = \mathbb{E}\left[d^{\mathrm{nc}}_{\mathrm{TMT}}|N\right]$. By Lemma 3, $\mathbb{E}[d^{\mathrm{nc}}_{\mathrm{TMT}}|N] = \delta_{\mathrm{TMT}}$. Therefore, $\mathbb{E}\left[d_{\mathrm{TMT}}|N\right] = \mathbb{E}\left[d^{\mathrm{pc}}_{\mathrm{TMT}}|N\right] = \delta_{\mathrm{TMT}}$.

A.7 Proof of Lemma 7

For the parental transmitted allele, A, under Assumption 3,

$$
\begin{aligned}
\mathbb{E}[A|N] &= \mathbb{P}(A = 1|N) \\
&= \mathbb{P}(A = 1|Z = 1, N)\mathbb{P}(Z = 1|N) + \mathbb{P}(A = 1|Z = 2, N)\mathbb{P}(Z = 2|N) \\
&= \frac{1}{2}\mathbb{P}(Z = 1|N) + \mathbb{P}(Z = 2|N) \\
&\to \frac{1}{2}\mathbb{P}(Z = 1) + \mathbb{P}(Z = 2)
\end{aligned}
$$

with probability 1 when $J \to \infty$. Notice that

$$
\begin{aligned}
\mathbb{E}[A] = \mathbb{P}(A = 1) &= \mathbb{P}(A = 1|Z = 1)\mathbb{P}(Z = 1) + \mathbb{P}(A = 1|Z = 2)\mathbb{P}(Z = 2) \\
&= \frac{1}{2}\mathbb{P}(Z = 1) + \mathbb{P}(Z = 2).
\end{aligned}
\tag{A7.a}
$$

Therefore, $\mathbb{E}[A|N] \to \mathbb{E}[A]$ with probability 1 when $J \to \infty$. Also,

$$
\begin{aligned}
\mathbb{E}[A_jA_k|N] &= \mathbb{P}(A_j = 1, A_k = 1|N) \\
&= \mathbb{P}(A_j = 1, A_k = 1|Z_j = 1, Z_k = 1, N)\mathbb{P}(Z_j = 1, Z_k = 1|N) \\
&\quad + \mathbb{P}(A_j = 1, A_k = 1|Z_j = 2, Z_k = 2, N)\mathbb{P}(Z_j = 2, Z_k = 2|N) \\
&\quad + \mathbb{P}(A_j = 1, A_k = 1|Z_j = 2, Z_k = 1, N)\mathbb{P}(Z_j = 2, Z_k = 1|N) \\
&\quad + \mathbb{P}(A_j = 1, A_k = 1|Z_j = 1, Z_k = 2, N)\mathbb{P}(Z_j = 1, Z_k = 2|N)
\end{aligned}
$$

$$
\begin{aligned}
&= \frac{1}{4}\mathbb{P}(Z_j = 1, Z_k = 1|N) + \mathbb{P}(Z_j = 2, Z_k = 2|N) \\
&\quad + \frac{1}{2}\mathbb{P}(Z_j = 2, Z_k = 1|N) + \frac{1}{2}\mathbb{P}(Z_j = 1, Z_k = 2|N) \\
&\to \frac{1}{4}\mathbb{P}(Z_j = 1, Z_k = 1) + \mathbb{P}(Z_j = 2, Z_k = 2) \\
&\quad + \frac{1}{2}\mathbb{P}(Z_j = 2, Z_k = 1) + \frac{1}{2}\mathbb{P}(Z_j = 1, Z_k = 2)
\end{aligned}
$$

with probability 1 when $J \to \infty$. Notice that

$$
\begin{aligned}
\mathbb{E}[A_j A_k] &= \mathbb{P}(A_j = 1, A_k = 1) \\
&= \mathbb{P}(A_j = 1, A_k = 1|Z_j = 1, Z_k = 1)\mathbb{P}(Z_j = 1, Z_k = 1) \\
&\quad + \mathbb{P}(A_j = 1, A_k = 1|Z_j = 2, Z_k = 2)\mathbb{P}(Z_j = 2, Z_k = 2) \\
&\quad + \mathbb{P}(A_j = 1, A_k = 1|Z_j = 2, Z_k = 1)\mathbb{P}(Z_j = 2, Z_k = 1) \\
&\quad + \mathbb{P}(A_j = 1, A_k = 1|Z_j = 1, Z_k = 2)\mathbb{P}(Z_j = 1, Z_k = 2) \\
&= \frac{1}{4}\mathbb{P}(Z_j = 1, Z_k = 1) + \mathbb{P}(Z_j = 2, Z_k = 2) \\
&\quad + \frac{1}{2}\mathbb{P}(Z_j = 2, Z_k = 1) + \frac{1}{2}\mathbb{P}(Z_j = 1, Z_k = 2). \qquad \text{(A7.b)}
\end{aligned}
$$

Then $\mathbb{E}[A_j A_k|N] \to \mathbb{E}[A_j A_k]$ with probability 1 as $J \to \infty$. So far we have shown:

$$
\mathbb{E}[A_j|N] \to \mathbb{E}[A_j], \qquad \mathbb{E}[A_k|N] \to \mathbb{E}[A_k], \qquad \mathbb{E}[A_j A_k|N] \to \mathbb{E}[A_j A_k],
$$

with probability 1 as $J \to \infty$. Then by Slutsky's Theorem,

$$
\mathbb{E}[A_j|N]\mathbb{E}[A_k|N] \to \mathbb{E}[A_j]\mathbb{E}[A_k]
$$

as $J \to \infty$. Together, we derive

$$
\begin{aligned}
\mathbb{C}(A_j, A_k|N) &= \mathbb{E}[A_j A_k|N] - \mathbb{E}[A_j|N]\mathbb{E}[A_k|N] \\
&\qquad \to \mathbb{E}[A_j A_k] - \mathbb{E}[A_j]\mathbb{E}[A_k] = \mathbb{C}(A_j, A_k)
\end{aligned}
$$

as $J \to \infty$. Now we show $\mathbb{C}(A_j, A_k) \geq 0$. By Equation (A7.a) and Equation (A7.b),

$$
\begin{aligned}
\mathbb{C}(A_j, A_k) &= \mathbb{E}[A_j A_k] - \mathbb{E}[A_j]\mathbb{E}[A_k] \\
&= \begin{bmatrix} \frac{1}{4}\mathbb{P}(Z_j = 1, Z_k = 1) + \mathbb{P}(Z_j = 2, Z_k = 2) \\ +\frac{1}{2}\mathbb{P}(Z_j = 2, Z_k = 1) + \frac{1}{2}\mathbb{P}(Z_j = 1, Z_k = 2) \end{bmatrix}
\end{aligned}
$$

$$
\begin{aligned}
&- \Big[\frac{1}{2}\mathbb{P}(Z_j = 1) + \mathbb{P}(Z_j = 2)\Big]\Big[\frac{1}{2}\mathbb{P}(Z_k = 1) + \mathbb{P}(Z_k = 2)\Big] \\
&= \frac{1}{4}\Big[\begin{array}{l} \mathbb{P}(Z_j = 1, Z_k = 1) + 4\mathbb{P}(Z_j = 2, Z_k = 2) \\ \quad +2\mathbb{P}(Z_j = 2, Z_k = 1) + 2\mathbb{P}(Z_j = 1, Z_k = 2) \end{array}\Big] \\
&\quad - \frac{1}{4}\Big[\mathbb{P}(Z_j = 1) + 2\mathbb{P}(Z_j = 2)\Big]\Big[\mathbb{P}(Z_k = 1) + 2\mathbb{P}(Z_k = 2)\Big] \\
&= \frac{1}{4}\mathbb{E}[Z_j Z_k] - \frac{1}{4}\mathbb{E}[Z_j]\mathbb{E}[Z_k] \\
&= \frac{1}{4}\mathbb{C}(Z_j, Z_k) \geq 0.
\end{aligned}
$$

A.8 Proof of Lemma 8

Let $W_{0j} = W_{0j}^m + W_{0j}^p$ and $W_{1j} = W_{1j}^m + W_{1j}^p$ where

$$
\begin{aligned}
W_{0j}^m &= \mathcal{I}(A_j^m = 0, Z_j^m = 1), \quad W_{0j}^p = \mathcal{I}(A_j^p = 0, Z_j^p = 1), \\
W_{1j}^m &= \mathcal{I}(A_j^m = 1, Z_j^m = 1), \quad W_{1j}^p = \mathcal{I}(A_j^p = 1, Z_j^p = 1).
\end{aligned}
$$

Then

$$
\begin{aligned}
\mathbb{C}(D_j, D_k | N) &= \mathbb{C}\left(\begin{array}{l} \big(W_{1j}^m + W_{1j}^p - W_{0j}^m - W_{0j}^p\big)\big(Y_j - \mu_c\big), \\ \big(W_{1k}^m + W_{1k}^p - W_{0k}^m - W_{0k}^p\big)\big(Y_k - \mu_c\big) \end{array} \middle| N \right) \\
&= \mathbb{C}\Big(\big(W_{1j}^m - W_{0j}^m\big)\big(Y_j - \mu_c\big), \big(W_{1k}^m - W_{0k}^m\big)\big(Y_k - \mu_c\big)\Big| N\Big) \\
&\quad + \mathbb{C}\Big(\big(W_{1j}^p - W_{0j}^p\big)\big(Y_j - \mu_c\big), \big(W_{1k}^p - W_{0k}^p\big)\big(Y_k - \mu_c\big)\Big| N\Big) \\
&\quad + \mathbb{C}\Big(\big(W_{1j}^m - W_{0j}^m\big)\big(Y_j - \mu_c\big), \big(W_{1k}^p - W_{0k}^p\big)\big(Y_k - \mu_c\big)\Big| N\Big) \\
&\quad + \mathbb{C}\Big(\big(W_{1j}^p - W_{0j}^p\big)\big(Y_j - \mu_c\big), \big(W_{1k}^m - W_{0k}^m\big)\big(Y_k - \mu_c\big)\Big| N\Big). \qquad \text{(A8.a)}
\end{aligned}
$$

By Eq. (A8.a), $\mathbb{C}(D_j, D_k|N)$ consists of four covariances whose calculations follow the same algebra. For the first covariance,

$$
\begin{aligned}
&\mathbb{C}\Big(\big(W_{1j}^m - W_{0j}^m\big)\big(Y_j - \mu_c\big), \big(W_{1k}^m - W_{0k}^m\big)\big(Y_k - \mu_c\big)\Big| N\Big) \qquad \text{(A8.b)} \\
&= \mathbb{C}\Big(\Big[\begin{array}{l} \mathcal{I}(A_j^m = 1, Z_j^m = 1) \\ \quad -\mathcal{I}(A_j^m = 0, Z_j^m = 1) \end{array}\Big]\Big[\begin{array}{l} \big(Y_j^m(1) - \mu_c\big)\mathcal{I}(A_j^m = 1) \\ \quad +\big(Y_j^m(0) - \mu_c\big)\mathcal{I}(A_j^m = 0) \end{array}\Big], \\
&\qquad \Big[\begin{array}{l} \mathcal{I}(A_k^m = 1, Z_k^m = 1) \\ \quad -\mathcal{I}(A_k^m = 0, Z_k^m = 1) \end{array}\Big]\Big[\begin{array}{l} \big(Y_k^m(1) - \mu_c\big)\mathcal{I}(A_k^m = 1) \\ \quad +\big(Y_k^m(0) - \mu_c\big)\mathcal{I}(A_k^m = 0) \end{array}\Big]\Big| N\Big) \\
&= \mathbb{C}\Big(\big(Y_j^m(1) - \mu_c\big)\mathcal{I}(A_j^m = 1, Z_j^m = 1) - \big(Y_j^m(0) - \mu_c\big)\mathcal{I}(A_j^m = 0, Z_j^m = 1),
\end{aligned}
$$

$$\big(Y_k^m(1)-\mu_c\big)\mathcal{I}(A_k^m=1,Z_k^m=1)-\big(Y_k^m(0)-\mu_c\big)\mathcal{I}(A_k^m=0,Z_k^m=1)\Big|N\Big)$$

$$=\mathbb{C}\Big((Y_j^m(1)-\mu_c)\mathcal{I}(A_j^m=1,Z_j^m=1),(Y_k^m(1)-\mu_c)\mathcal{I}(A_k^m=1,Z_k^m=1)\Big|N\Big) \tag{A8.c}$$

$$+\mathbb{C}\Big(-(Y_j^m(0)-\mu_c)\mathcal{I}(A_j^m=0,Z_j^m=1),-(Y_k^m(0)-\mu_c)\mathcal{I}(A_k^m=0,Z_k^m=1)\Big|N\Big) \tag{A8.d}$$

$$+\mathbb{C}\Big((Y_j^m(1)-\mu_c)\mathcal{I}(A_j^m=1,Z_j^m=1),-(Y_k^m(0)-\mu_c)\mathcal{I}(A_k^m=0,Z_k^m=1)\Big|N\Big) \tag{A8.e}$$

$$+\mathbb{C}\Big(-(Y_j^m(0)-\mu_c)\mathcal{I}(A_j^m=0,Z_j^m=1),(Y_k^m(1)-\mu_c)\mathcal{I}(A_k^m=1,Z_k^m=1)\Big|N\Big). \tag{A8.f}$$

We first calculate Eq. (A8.c) as follows. Calculations for the other three covariances should follow a similar approach.

$$\begin{aligned}
\text{Eq. (A8.c)} &= \mathbb{E}\left[\big(Y_j^m(1)-\mu_c\big)\mathcal{I}(A_j^m=1,Z_j^m=1)\big(Y_k^m(1)-\mu_c\big)\mathcal{I}(A_k^m=1,Z_k^m=1)\Big|N\right]\\
&\quad-\mathbb{E}\left[\big(Y_j^m(1)-\mu_c\big)\mathcal{I}(A_j^m=1,Z_j^m=1)\Big|N\right]\cdot\mathbb{E}\left[\big(Y_k^m(1)-\mu_c\big)\mathcal{I}(A_k^m=1,Z_k^m=1)\Big|N\right]\\
&= \mathbb{E}\left[\big(Y_j^m(1)-\mu_c\big)\big(Y_k^m(1)-\mu_c\big)\Big|\begin{array}{l} A_j^m=A_k^m=1,\\ Z_j^m=Z_k^m=1,N\end{array}\right]\mathbb{P}\Big(\begin{array}{l} A_j^m=A_k^m=1,\\ Z_j^m=Z_k^m=1\end{array}\Big|N\Big)\\
&\quad-\mathbb{E}[Y_j^m(1)-\mu_c|A_j^m=1,Z_j^m=1,N]\mathbb{P}(A_j^m=1,Z_j^m=1|N)\\
&\qquad\times\mathbb{E}[Y_k^m(1)-\mu_c|A_k^m=1,Z_k^m=1,N]\mathbb{P}(A_k^m=1,Z_k^m=1|N)\\
&= \mathbb{E}\left[\big(Y_j^m(1)-\mu_c\big)\big(Y_k^m(1)-\mu_c\big)\Big|\begin{array}{l} A_j^m=A_k^m=1,\\ Z_j^m=Z_k^m=1,N\end{array}\right]\frac{1}{4}\mathbb{P}(Z_j^m=Z_k^m=1|N)\\
&\quad-\mathbb{E}[Y_j^m(1)-\mu_c|A_j^m=1,Z_j^m=1,N]\frac{1}{2}\mathbb{P}(Z_j^m=1|N)\\
&\qquad\times\mathbb{E}[Y_k^m(1)-\mu_c|A_k^m=1,Z_k^m=1,N]\frac{1}{2}\mathbb{P}(Z_k^m=1|N)
\end{aligned}$$

Since Lemma 2 implies $Y^m(1)\perp\!\!\!\perp A^m|Z^m=1$, then the above equation can be further derived as follows.

$$\begin{aligned}
\text{Eq. (A8.c)} &= \frac{1}{4}\mathbb{E}\left[\big(Y_j^m(1)-\mu_c\big)\big(Y_k^m(1)-\mu_c\big)\Big|Z_j^m=Z_k^m=1,N\right]\mathbb{P}(Z_j^m=Z_k^m=1|N)\\
&\quad-\frac{1}{4}\mathbb{E}\left[Y_j^m(1)-\mu_c\Big|Z_j^m=1,N\right]\mathbb{P}(Z_j^m=1|N)\cdot\mathbb{E}\left[Y_k^m(1)-\mu_c\Big|Z_k^m=1,N\right]\mathbb{P}(Z_k^m=1|N)\\
&= \frac{1}{4}\mathbb{E}\left[\big(Y_j^m(1)-\mu_c\big)\big(Y_k^m(1)-\mu_c\big)\mathcal{I}(Z_j^m=Z_k^m=1)\Big|N\right]\\
&\quad-\frac{1}{4}\mathbb{E}\left[\big(Y_j^m(1)-\mu_c\big)\mathcal{I}(Z_j^m=1)\Big|N\right]\cdot\mathbb{E}\left[\big(Y_k^m(1)-\mu_c\big)\mathcal{I}(Z_k^m=1)\Big|N\right]
\end{aligned}$$

$$= \frac{1}{4}\mathbb{C}\left(\big(Y_j^m(1)-\mu_c\big)\mathcal{I}(Z_j^m=1), \big(Y_k^m(1)-\mu_c\big)\mathcal{I}(Z_k^m=1)\Big|N\right)$$

Similarly, we can derive the following equations.

$$\begin{aligned}
\text{Eq. (A8.d)} &= \frac{1}{4}\mathbb{C}\left(-\big(Y_j^m(0)-\mu_c\big)\mathcal{I}(Z_j^m=1), -\big(Y_k^m(0)-\mu_c\big)\mathcal{I}(Z_k^m=1)\Big|N\right)\\
\text{Eq. (A8.e)} &= \frac{1}{4}\mathbb{C}\left(\big(Y_j^m(1)-\mu_c\big)\mathcal{I}(Z_j^m=1), -\big(Y_k^m(0)-\mu_c\big)\mathcal{I}(Z_k^m=1)\Big|N\right)\\
\text{Eq. (A8.f)} &= \frac{1}{4}\mathbb{C}\left(-\big(Y_j^m(0)-\mu_c\big)\mathcal{I}(Z_j^m=1), \big(Y_k^m(1)-\mu_c\big)\mathcal{I}(Z_k^m=1)\Big|N\right)
\end{aligned}$$

Plugging into Eq. (A8.b) implies the following results.

$$\begin{aligned}
&\mathbb{C}\Big(\big(W_{1j}^m-W_{0j}^m\big)\big(Y_j-\mu_c\big), \big(W_{1k}^m-W_{0k}^m\big)\big(Y_k-\mu_c\big)\Big|N\Big)\\
&= \text{Eq. (A8.c)} + \text{Eq. (A8.d)} + \text{Eq. (A8.e)} + \text{Eq. (A8.f)}\\
&= \frac{1}{4}\mathbb{C}\left(\begin{array}{c}\big(Y_j^m(1)-\mu_c\big)\mathcal{I}(Z_j^m=1)-\big(Y_j^m(0)-\mu_c\big)\mathcal{I}(Z_j^m=1),\\ \big(Y_k^m(1)-\mu_c\big)\mathcal{I}(Z_k^m=1)-\big(Y_k^m(0)-\mu_c\big)\mathcal{I}(Z_k^m=1)\end{array}\Big|N\right)\\
&= \frac{1}{4}\mathbb{C}\left(\big(Y_j^m(1)-Y_j^m(0)\big)\mathcal{I}(Z_j^m=1), \big(Y_k^m(1)-Y_k^m(0)\big)\mathcal{I}(Z_k^m=1)\Big|N\right)\\
&= \frac{1}{4}\mathbb{E}\left[\mathbb{C}\big(Y_j^m(1)-Y_j^m(0), Y_k^m(1)-Y_k^m(0)\big|Z_j^m=Z_k^m=1,N\big)\Big|N\right]\\
&\qquad + \frac{1}{4}\mathbb{C}\left(\begin{array}{c}\mathbb{E}[Y_j^m(1)-Y_j^m(0)|Z_j^m=Z_k^m=1,N],\\ \mathbb{E}[Y_k^m(1)-Y_k^m(0)|Z_j^m=Z_k^m=1,N]\end{array}\Big|N\right) \qquad \text{(by The Law of Total Covariance)}\\
&= \frac{1}{4}\mathbb{E}\left[\mathbb{C}\big((\alpha_2-2\alpha_1+\alpha_0)A_j^p+(\alpha_1-\alpha_0), (\alpha_2-2\alpha_1+\alpha_0)A_k^p+(\alpha_1-\alpha_0)\big|Z_j^m=Z_k^m=1,N\big)\Big|N\right]\\
&\qquad + \frac{1}{4}\mathbb{C}\left(\begin{array}{c}\mathbb{E}[(\alpha_2-2\alpha_1+\alpha_0)A_j^p+(\alpha_1-\alpha_0)|Z_j^m=Z_k^m=1,N],\\ \mathbb{E}[(\alpha_2-2\alpha_1+\alpha_0)A_k^p+(\alpha_1-\alpha_0)|Z_j^m=Z_k^m=1,N]\end{array}\Big|N\right) \qquad \text{(by Eq. (2))}\\
&= \frac{1}{4}\mathbb{E}\left[(\alpha_2-2\alpha_1+\alpha_0)^2\mathbb{C}(A_j^p, A_k^p|Z_j^m=Z_k^m=1,N)\Big|N\right]\\
&\qquad + \frac{1}{4}\mathbb{C}\left(\begin{array}{c}(\alpha_2-2\alpha_1+\alpha_0)\mathbb{E}[A_j^p|Z_j^m=Z_k^m=1,N]+(\alpha_1-\alpha_0),\\ (\alpha_2-2\alpha_1+\alpha_0)\mathbb{E}[A_k^p|Z_j^m=Z_k^m=1,N]+(\alpha_1-\alpha_0)\end{array}\Big|N\right)\\
&= \frac{(\alpha_2-2\alpha_1+\alpha_0)^2}{4}\mathbb{E}\left[\mathbb{C}(A_j^p, A_k^p|Z_j^m=Z_k^m=1,N)\Big|N\right]\\
&\qquad + \frac{(\alpha_2-2\alpha_1+\alpha_0)^2}{4}\mathbb{C}\left(\mathbb{E}[A_j^p|Z_j^m=Z_k^m=1,N], \mathbb{E}[A_k^p|Z_j^m=Z_k^m=1,N]\Big|N\right)\\
&= \frac{(\alpha_2-2\alpha_1+\alpha_0)^2}{4}\mathbb{C}\left(A_j^p, A_k^p|N\right) \qquad \text{(by The Law of Total Covariance)}
\end{aligned}$$

Similarly, we can calculate the other three covariances in Eq. (A8.b) and plug all four covariances into Eq. (A8.b) to derive the following equation.

$$\begin{aligned}
\mathbb{C}(D_j, D_k|N) &= \frac{(\alpha_2 - 2\alpha_1 + \alpha_0)^2}{4}\mathbb{C}\left(A_j^p, A_k^p|N\right) \\
&\quad + \frac{(\alpha_2 - 2\alpha_1 + \alpha_0)^2}{4}\mathbb{C}\left(A_j^m, A_k^m|N\right) \\
&\quad + \frac{(\alpha_2 - 2\alpha_1 + \alpha_0)^2}{4}\mathbb{C}\left(A_j^p, A_k^m|N\right) \\
&\quad + \frac{(\alpha_2 - 2\alpha_1 + \alpha_0)^2}{4}\mathbb{C}\left(A_j^m, A_k^p|N\right) \\
&= \frac{(\alpha_2 - 2\alpha_1 + \alpha_0)^2}{4}\left(\begin{array}{c}\mathbb{C}(A_j^p, A_k^p|N) + \mathbb{C}(A_j^m, A_k^m|N) \\ +\mathbb{C}(A_j^p, A_k^m|N) + \mathbb{C}(A_j^m, A_k^p|N)\end{array}\right) \qquad \text{(A8.g)}
\end{aligned}$$

When $\delta_{\text{TMT}} = 0$, Theorem 1 implies that $\alpha_0 = \alpha_1 = \alpha_2$, and thereby, $(\alpha_2 - 2\alpha_1 + \alpha_0)^2 = 0$, Eq. (A8.g) equals 0. Therefore, $\mathbb{C}(D_j, D_k|N) = 0$ for $j, k \in \mathcal{J}$ and $j \neq k$ and when $\delta_{\text{TMT}} = 0$.

To show non-negative asymptotic value of Eq. (A8.b) when $\delta_{\text{TMT}} \neq 0$, by Lemma 7, when $J \to \infty$, $\mathbb{C}(A_j, A_k|N) \to \mathbb{C}(A_j, A_k) \geq 0$. Therefore,

$$\lim_{J\to\infty} \mathbb{C}(D_j, D_k|N) = \frac{(\alpha_2 - 2\alpha_1 + \alpha_0)^2}{4}\left(\begin{array}{c}\mathbb{C}(A_j^p, A_k^p) + \mathbb{C}(A_j^m, A_k^m) \\ +\mathbb{C}(A_j^p, A_k^m) + \mathbb{C}(A_j^m, A_k^p)\end{array}\right) \geq 0.$$

The equality, $\lim_{J\to\infty} \mathbb{C}(D_j, D_k|N) = 0$, holds for the following scenarios:

(A) Under a true null hypothesis where $\alpha_0 = \alpha_1 = \alpha_2$ so that $(\alpha_2 - 2\alpha_1 + \alpha_0) = 0$.

(B) Under a true alternative hypothesis where $\alpha_1 - \alpha_0 = \alpha_2 - \alpha_1$ so that $(\alpha_2 - 2\alpha_1 + \alpha_0) = 0$. This setting is equivalent to an additive genetic effect model.

(C) The genotypes of the jth and the kth children have zero covariance.

A.9 Proof of Lemma 9

Let $D_j = (W_{1j} - W_{0j})(Y_j - \mu_c)$ and $\mathcal{T} = \{\mathcal{T}_0, \mathcal{T}_1, \mathcal{T}_{00}, \mathcal{T}_{11}\}$. Then $\mathbb{V}(d_{\text{TMT}}^{\text{pc}}|N)$ equals

$$\begin{aligned}
&\mathbb{V}\left(\frac{2}{N}\sum_{j\in\mathcal{J}}(W_{1j} - W_{0j})(Y_j - \mu_c)\middle| N\right) \\
&= \frac{4}{N^2}\left(\sum_{j\in\mathcal{J}}\mathbb{V}(D_j|N) + \sum_{j\neq k\in\mathcal{J}}\mathbb{C}(D_j, D_k|N)\right)
\end{aligned}$$

$$
\begin{aligned}
&= \frac{4}{N^2}\sum_{j\in\mathcal{J}}\Big(\mathbb{E}\big[\mathbb{V}(D_j|\mathcal{T},N)\big|N\big] \;+\mathbb{V}\big(\mathbb{E}[D_j|\mathcal{T},N]\big|N\big)\Big) + \frac{4}{N^2}\sum_{j\neq k\in\mathcal{J}}\mathbb{C}(D_j,D_k|N)\\
&= \frac{4}{N^2}\sum_{j\in\mathcal{J}}\mathbb{E}\Big[\mathbb{V}\big((W_{1j}-W_{0j})(Y_j-\mu_c)\big|\mathcal{T},N\big)\Big|N\Big]\\
&\quad + \frac{4}{N^2}\sum_{j\in\mathcal{J}}\mathbb{V}\Big(\mathbb{E}\big[(W_{1j}-W_{0j})(Y_j-\mu_c)\big|\mathcal{T},N\big]\Big|N\Big)\\
&\quad + \frac{4}{N^2}\sum_{j\neq k\in\mathcal{J}}\mathbb{C}(D_j,D_k|N)\\
&= \frac{4}{N^2}\sum_{j\in\mathcal{J}}\Big(\frac{|\mathcal{T}_{11}|}{J}\mathbb{V}(2Y|\mathcal{T}_{11}) + \frac{|\mathcal{T}_{00}|}{J}\mathbb{V}(2Y|\mathcal{T}_{00}) + \frac{|\mathcal{T}_{1}|}{J}\mathbb{V}(Y|\mathcal{T}_{1}) + \frac{|\mathcal{T}_{0}|}{J}\mathbb{V}(Y|\mathcal{T}_{0})\Big)\\
&\quad + \frac{4}{N^2}\sum_{j\in\mathcal{J}}\mathbb{V}\Big(\frac{|\mathcal{T}_{11}|}{J}(\mu_{11}-2\mu_c) + \frac{|\mathcal{T}_{00}|}{J}(2\mu_c-\mu_{00}) + \frac{|\mathcal{T}_{1}|}{J}(\mu_1-\mu_c) + \frac{|\mathcal{T}_{0}|}{J}(\mu_c-\mu_0)\Big)\\
&\quad + \frac{4}{N^2}\sum_{j\neq k\in\mathcal{J}}\mathbb{C}(D_j,D_k|N)\\
&= \frac{4}{N^2}\Big(|\mathcal{T}_{11}|\mathbb{V}(2Y|\mathcal{T}_{11}) + |\mathcal{T}_{1}|\mathbb{V}(Y|\mathcal{T}_{1}) + |\mathcal{T}_{00}|\mathbb{V}(2Y|\mathcal{T}_{00}) + |\mathcal{T}_{0}|\mathbb{V}(Y|\mathcal{T}_{0})\Big)\\
&\quad + \frac{4}{N^2J}\mathbb{V}\Big(|\mathcal{T}_{11}|(\mu_{11}-2\mu_c) + |\mathcal{T}_{1}|(\mu_1-\mu_c) + |\mathcal{T}_{00}|(2\mu_c-\mu_{00}) + |\mathcal{T}_{0}|(\mu_c-\mu_0)\Big)\\
&\quad + \frac{4}{N^2}\sum_{j\neq k\in\mathcal{J}}\mathbb{C}(D_j,D_k|N).
\end{aligned}
$$

Let

$$
\begin{aligned}
\sigma^2_{\text{pc},1} &= \frac{4}{N^2}\Big(|\mathcal{T}_{11}|\mathbb{V}(2Y|\mathcal{T}_{11}) + |\mathcal{T}_{1}|\mathbb{V}(Y|\mathcal{T}_{1}) + |\mathcal{T}_{00}|\mathbb{V}(2Y|\mathcal{T}_{00}) + |\mathcal{T}_{0}|\mathbb{V}(Y|\mathcal{T}_{0})\Big),\\
\sigma^2_{\text{pc},2} &= \frac{4}{N^2J}\mathbb{V}\Big(|\mathcal{T}_{11}|(\mu_{11}-2\mu_c) + |\mathcal{T}_{1}|(\mu_1-\mu_c) + |\mathcal{T}_{00}|(2\mu_c-\mu_{00}) + |\mathcal{T}_{0}|(\mu_c-\mu_0)\Big),\\
\sigma^2_{\text{pc},3} &= \frac{4}{N^2}\sum_{j\neq k\in\mathcal{J}}\mathbb{C}(D_j,D_k|N).
\end{aligned}
$$

By Lemma 6, $\mathbb{E}\left[\hat{\sigma}^2_{\text{TMT}}\middle| N\right] = \sigma^2_{\text{pc},1}$. When the null hypothesis is true, then $\mu_1 = \mu_0 = \mu_c$ and $\mu_{11} = \mu_{00} = 2\mu_c$ so that $\sigma^2_{\text{pc},2} = 0$. Under a true alternative hypothesis, $\sigma^2_{\text{pc},2} > 0$. By Lemma 8, when the null hypothesis is true then $\sigma^2_{\text{pc},3} = 0$. When the alternative hypothesis is true $\sigma^2_{\text{pc},3}$ converges almost surely to a non-negative value as $J \to \infty$. Since $\mathbb{V}(d^{\text{pc}}_{\text{TMT}}|N) = \sigma^2_{\text{pc},1} + \sigma^2_{\text{pc},2} + \sigma^2_{\text{pc},3}$, it follows that:

(A) under null, $\hat{\sigma}^2_{\text{TMT}}$ is an unbiased estimator for $\mathbb{V}(d^{\text{pc}}_{\text{TMT}}|N)$;

(B) under alternative, $\hat{\sigma}^2_{\text{TMT}}$ underestimates $\mathbb{V}(d^{\text{pc}}_{\text{TMT}}|N)$ as $J \to \infty$.

As an aside,

$$\lim_{J\to\infty}\frac{\sigma^2_{\text{pc},2}}{\sigma^2_{\text{pc},1}}=\lim_{J\to\infty}\frac{1}{J}\frac{\mathbb{V}\big(|\mathcal{T}_{11}|(\mu_{11}-2\mu_c)+|\mathcal{T}_1|(\mu_1-\mu_c)+|\mathcal{T}_{00}|(2\mu_c-\mu_{00})+|\mathcal{T}_0|(\mu_c-\mu_0)\big)}{|\mathcal{T}_{11}|\mathbb{V}(2Y|\mathcal{T}_{11})+|\mathcal{T}_1|\mathbb{V}(Y|\mathcal{T}_1)+|\mathcal{T}_{00}|\mathbb{V}(2Y|\mathcal{T}_{00})+|\mathcal{T}_0|\mathbb{V}(Y|\mathcal{T}_0)}=0.$$

A.10 Proof of Lemma 10

Let $\mathbb{P}(Z=1)=\omega$. By Assumption 3[A], $N/(2J)\to\omega$ with probability 1 when $J\to\infty$. It is the case that

$$\frac{2J}{N}(W_{1j}-W_{0j})(Y_j-\hat{\mu}_c)$$

for $j\in\mathcal{J}$ are exchangeable with each term having finite first absolute moment. Given these properties, by Lemma 4 and Equation 2.2 of ref. [**kingman1978**] implies

$$\lim_{J\to\infty}d_{\text{TMT}}=\lim_{J\to\infty}\frac{1}{J}\sum_{j\in\mathcal{J}}\frac{2J}{N}(W_{1j}-W_{0j})(Y_j-\hat{\mu}_c)=\mathbb{E}\left[\frac{1}{\omega}(W_1-W_0)(Y-\mu_c)\right]$$

almost surely. Similarly,

$$\lim_{J\to\infty}d^{\text{pc}}_{\text{TMT}}=\lim_{J\to\infty}\frac{1}{J}\sum_{j\in\mathcal{J}}\frac{2J}{N}(W_{1j}-W_{0j})(Y_j-\mu_c)=\mathbb{E}\left[\frac{1}{\omega}(W_1-W_0)(Y-\mu_c)\right]$$

almost surely. Therefore,

$$\lim_{J\to\infty}d_{\text{TMT}}=\lim_{J\to\infty}d^{\text{pc}}_{\text{TMT}}$$

almost surely. Together with Lemma 5 where $\mathbb{E}[d_{\text{TMT}}|N]=\mathbb{E}[d^{\text{pc}}_{\text{TMT}}|N]=\delta_{\text{TMT}}$, by the Continuous Mapping Theorem,

$$\lim_{J\to\infty}\mathbb{V}(d_{\text{TMT}}|N)=\lim_{J\to\infty}\mathbb{V}(d^{\text{pc}}_{\text{TMT}}|N)$$

almost surely. In other words, $|\mathbb{V}(d^{\text{pc}}_{\text{TMT}}|N)-\mathbb{V}(d_{\text{TMT}}|N)|\to 0$ with probability 1 as $J\to\infty$.

A.11 Proof of Lemma 11

$$\begin{aligned}\delta_{\text{TDT}}=&\ \mathbb{P}(Y=1|Z^m=1,N)\mathbb{E}[\mathcal{I}(A^m=1)-\mathcal{I}(A^m=0)|Y=1,Z^m=1,N]\\&+\mathbb{P}(Y=1|Z^p=1,N)\mathbb{E}[\mathcal{I}(A^p=1)-\mathcal{I}(A^p=0)|Y=1,Z^p=1,N]\end{aligned}$$

$$
\begin{aligned}
&= \mathbb{P}(Y=1|Z^m=1,N)\mathbb{E}[\mathcal{I}(A^m=1)|Y=1,Z^m=1,N] \\
&\quad - \mathbb{P}(Y=1|Z^m=1,N)\mathbb{E}[\mathcal{I}(A^m=0)|Y=1,Z^m=1,N] \\
&\quad + \mathbb{P}(Y=1|Z^p=1,N)\mathbb{E}[\mathcal{I}(A^p=1)|Y=1,Z^p=1,N] \\
&\quad - \mathbb{P}(Y=1|Z^p=1,N)\mathbb{E}[\mathcal{I}(A^p=0)|Y=1,Z^p=1,N] \\
&= \mathbb{P}(Y=1|Z^m=1,N)\mathbb{P}(A^m=1|Y=1,Z^m=1,N) \\
&\quad - \mathbb{P}(Y=1|Z^m=1,N)\mathbb{P}(A^m=0|Y=1,Z^m=1,N) \\
&\quad + \mathbb{P}(Y=1|Z^p=1,N)\mathbb{P}(A^p=1|Y=1,Z^p=1,N) \\
&\quad - \mathbb{P}(Y=1|Z^p=1,N)\mathbb{P}(A^p=0|Y=1,Z^p=1,N) \\
&= \mathbb{P}(Y=1,A^m=1|Z^m=1,N) - \mathbb{P}(Y=1,A^m=0|Z^m=1,N) \\
&\quad + \mathbb{P}(Y=1,A^p=1|Z^p=1,N) - \mathbb{P}(Y=1,A^p=0|Z^p=1,N) \\
&= \mathbb{E}\Big[\mathcal{I}(Y=1,A^m=1) - \mathcal{I}(Y=1,A^m=0)\Big|Z^m=1,N\Big] \\
&\quad + \mathbb{E}\Big[\mathcal{I}(Y=1,A^p=1) - \mathcal{I}(Y=1,A^p=0)\Big|Z^p=1,N\Big] \\
&= \mathbb{E}\Big[Y\mathcal{I}(A^m=1) - Y\mathcal{I}(A^m=0)\Big|Z^m=1,N\Big] \\
&\quad + \mathbb{E}\Big[Y\mathcal{I}(A^p=1) - Y\mathcal{I}(A^p=0)\Big|Z^p=1,N\Big] \\
&= \mathbb{E}\Big[\mathcal{I}(A^m=1)Y^m(1) - \mathcal{I}(A^m=0)Y^m(0)\Big|Z^m=1,N\Big] \\
&\quad + \mathbb{E}\Big[\mathcal{I}(A^p=1)Y^p(1) - \mathcal{I}(A^p=0)Y^p(0)\Big|Z^p=1,N\Big] \\
&= \mathbb{P}(A^m=1|Z^m=1,N)\mathbb{E}[Y^m(1)|Z^m=1,N] \\
&\quad - \mathbb{P}(A^m=0|Z^m=1,N)\mathbb{E}[Y^m(0)|Z^m=1,N] \\
&\quad + \mathbb{P}(A^p=1|Z^p=1,N)\mathbb{E}[Y^p(1)|Z^p=1,N] \\
&\quad - \mathbb{P}(A^p=0|Z^p=1,N)\mathbb{E}[Y^p(0)|Z^p=1,N] \\
&= \frac{1}{2}\mathbb{E}[Y^m(1)-Y^m(0)|Z^m=1,N] + \frac{1}{2}\mathbb{E}[Y^p(1)-Y^p(0)|Z^p=1,N] \\
&= \delta_{\mathrm{TMT}}
\end{aligned}
$$

A.12 Proof of Lemma 12

$$
\begin{aligned}
\mathbb{E}[d_{\mathrm{TDT}}|N] &= \mathbb{E}\Big[\frac{2}{N}\sum_{j\in\mathcal{J}}(W_{1j}-W_{0j})Y_j\Big|N\Big] \\
&= \frac{2}{N}\mathbb{E}\Big[\sum_{j\in\mathcal{J}}(2A_j^m-1)Y_j\mathcal{I}(Z_j^m=1) + (2A_j^p-1)Y_j\mathcal{I}(Z_j^p=1)\Big|N\Big]
\end{aligned}
$$

$$
\begin{aligned}
&= \frac{2J}{N}\mathbb{E}\Big[(2A^m-1)\mathcal{I}(Y=1,Z^m=1)+(2A^p-1)\mathcal{I}(Y=1,Z^p=1)\Big|N\Big] \\
&= \frac{2J}{N}\mathbb{P}(Y=1,Z^m=1|N)\mathbb{E}[2A^m-1|Y=1,Z^m=1,N] \\
&\quad + \frac{2J}{N}\mathbb{P}(Y=1,Z^p=1|N)\mathbb{E}[2A^p-1|Y=1,Z^p=1,N] \\
&= \frac{2J}{N}\mathbb{P}(Z^m=1|N)\mathbb{P}(Y=1|Z^m=1,N)\mathbb{E}[2A^m-1|Y=1,Z^m=1,N] \\
&\quad + \frac{2J}{N}\mathbb{P}(Z^p=1|N)\mathbb{P}(Y=1|Z^p=1,N)\mathbb{E}[2A^p-1|Y=1,Z^p=1,N] \qquad \text{(A12.a)} \\
&= \frac{2J}{N}\frac{N}{2J}\mathbb{P}(Y=1|Z^m=1,N)\mathbb{E}[2A^m-1|Y=1,Z^m=1,N] \\
&\quad + \frac{2J}{N}\frac{N}{2J}\mathbb{P}(Y=1|Z^p=1,N)\mathbb{E}[2A^p-1|Y=1,Z^p=1,N] \qquad \text{(A12.b)} \\
&= \mathbb{P}(Y=1|Z^m=1,N)\mathbb{E}[2A^m-1|Y=1,Z^m=1,N] \\
&\quad + \mathbb{P}(Y=1|Z^p=1,N)\mathbb{E}[2A^p-1|Y=1,Z^p=1,N] \\
&= \eta\mathbb{E}[\mathcal{I}(A^m=1)-\mathcal{I}(A^m=0)|Y=1,Z^m=1,N] \\
&\quad + \eta\mathbb{E}[\mathcal{I}(A^p=1)-\mathcal{I}(A^p=0)|Y=1,Z^p=1,N] \\
&= \delta_{\text{TDT}}
\end{aligned}
$$

Line (A12.a) to line (A12.b) is due to the fact that $\mathbb{P}(Z_j^m=1|N)=\mathbb{P}(Z_j^p=1|N)=N/(2J)$.

A.13 Proof of Lemma 13

By Theorem 1, $\delta_{\text{TMT}} \neq 0$ at marker d if and only if $\text{ACE}(G_d \to Y) \neq 0$. By Theorem 2, $\delta_{\text{TDT}} \neq 0$ at marker d if and only if $\text{ACE}(G_d \to Y) \neq 0$. Then by Lemma 1, $\text{ACE}(G_d \to Y) \neq 0$ if and only if $\text{ACE}(A_d^m \to Y) + \text{ACE}(A_d^p \to Y) \neq 0$, which is equivalent to either $\text{ACE}(A_d^m \to Y) \neq 0$ or $\text{ACE}(A_d^p \to Y) \neq 0$ under Assumption 1. This means either $A_d^m \to Y$ or $A_d^p \to Y$ according to Definition 3. This further implies that either $A_d^m \hookrightarrow Y$ or $A_d^p \hookrightarrow Y$ by Definition 11 since marker d is in complete linkage with itself, which means $(A_d^m, A_d^p) \hookrightarrow Y$ by Definition 12.

A.14 Relationship to the direct-indirect trio model

The “direct-indirect” trio model [2, 3] assumes the following for a quantitative trait and a single SNP (using our notation):

$$Y_j = \beta_0 + \beta_1 G_j + \beta_2 Z_j^m + \beta_3 Z_j^p + \epsilon_j.$$

The parameters $\beta_0, \ldots, \beta_3$ are fixed effects, where sometimes β_2 and β_3 are combined into a single parameter. The association parameter β_1 is called a direct effect and the parameters β_2 and β_3 are called indirect effects. The assumptions on the random variable depend on the particular article, but it is possible to model $\epsilon_j | G_j, Z_j^m, Z_j^p$ according to the standard linear mixed effects model that takes into account genetic relatedness among the children.

If we restrict γ_j from our model Eq. (1) to be

$$\gamma_j = \beta_0 + \beta_2 Z_j^m + \beta_3 Z_j^p + \epsilon_j$$

and assume $\beta_1 = \alpha_1 - \alpha_0 = \alpha_2 - \alpha_1$ (thereby restricting $\alpha_0 \mathcal{I}(G_j = 0) + \alpha_1 \mathcal{I}(G_j = 1) + \alpha_2 \mathcal{I}(G_j = 2)$ to be equal to $\beta_1 G_j$), then it can be seen that the direct-indirect trio model is a restricted form of our model. We do not require γ_j to be of this form and we make more general assumptions about the random term.

It has been shown that the direct-indirect trio model is susceptible to various forms of confounding and is not a test of causality [4, 5]. However, if one applies the TMT to a parent-child trio study and verifies Assumption 2 that $\mathbb{P}(A = a | Z = 1) = \mathbb{P}(A = a | Z = 1, \gamma)$ for $a \in \{0, 1\}$, then a potential outcomes based causal inference on the parameter β_2 (or our parameters $\alpha_0, \alpha_1, \alpha_2$) can be made. The trade-off is that the TMT is performed on families with at least one heterozygote parent, whereas the direct-indirect association model can be fit to all families. It estimates parental coefficients, whereas the TMT is not designed to do so. Therefore, these two models can be used in conjunction with one another if one assumes the restricted direct-indirect model form is true. The direct-indirect model provides parameter estimates for all three terms, but is limited to conclusions about association. The TMT rigorously and robustly estimates the causal effect within the child, and it allows for stronger conclusions regarding causality.

B Simulations

B.1 Simulating trio genotypes

Both parental genotypes matrices $\boldsymbol{Z}^m$ and $\boldsymbol{Z}^p$ were sampled from a structured population based on a standard admixture model [1, 6–8] with $K = 4$ admixed populations. We configured this population to have $F_{\mathrm{ST}} = 0.2$ by utilizing the `bnpsd` R package [1, 9]. We simulated the ancestral allele frequencies from the Uniform$(0.1, 0.9)$, which is an option in the `bnpsd` R package. To visualize the population structure, we presented the co-ancestry coefficients between individuals from the structured population in Figure S2.

B.2 Simulating quantitative trait

We first generated a non-genetic factor associated with the population structure. Let $\boldsymbol{E} = \{E_j\}$ be the random non-genetic factor. We adopted the admixture proportion $\boldsymbol{q} = \{q_{ju}\}$ from Appendix B.1 to simulate E_j as

$$E_j = \sum_{u=1}^{K} q_{ju} R_u, \quad R_u \sim \mathcal{N}(u, 1) \text{ for } u \in [1 : K], \quad K = 4.$$

Let $\mathcal{C}$ be the loci indices set for all causal SNPs. We followed Equation (13) to simulate the child's trait $\boldsymbol{Y} = \{Y_j\}$ as

$$Y_j = \iota + \sum_{i \in \mathcal{C}} \Big(\alpha_0 \mathcal{I}(G_{ij} = 0) + \alpha_1 \mathcal{I}(G_{ij} = 1) + \alpha_2 \mathcal{I}(G_{ij} = 2) \Big) + \beta E_j + \epsilon_j.$$

To show that the above equation satisfies the trait model in Equation (1), at the cth causal SNP ($c \in \mathcal{C}$), rewrite the equation as

$$\begin{aligned} Y_j &= \alpha_0 \mathcal{I}(G_{cj} = 0) + \alpha_1 \mathcal{I}(G_{cj} = 1) + \alpha_2 \mathcal{I}(G_{cj} = 2) + \gamma_j, \\ \gamma_j &= \iota + \zeta_j + \beta E_j + \epsilon_j, \\ \zeta_j &= \sum_{i \in \mathcal{C}, i \neq c} \Big(\alpha_0 \mathcal{I}(G_{ij} = 0) + \alpha_1 \mathcal{I}(G_{ij} = 1) + \alpha_2 \mathcal{I}(G_{ij} = 2) \Big), \end{aligned}$$

where the first line satisfies Equation (1). We considered three sets of $(\alpha_0, \alpha_1, \alpha_2)$ as follows

$$\begin{aligned} &\text{Set A:} \quad \alpha_0 = \beta, \alpha_1 = 2\beta, \alpha_2 = 3\beta; \\ &\text{Set B:} \quad \alpha_0 = \beta, \alpha_1 = 2\beta, \alpha_2 = 7\beta; \end{aligned}$$

$$\text{Set C:} \quad \alpha_0 = \beta, \alpha_1 = 6\beta, \alpha_2 = 7\beta.$$

Then the ratio $(\alpha_2 - \alpha_1)/(\alpha_1 - \alpha_0)$ is 1, 5 and 1/5 for the above Set A, B and C. Given the heritability $h^2 = \sigma_a^2/(\sigma_a^2 + \sigma_e^2)$ where we set $\sigma_e^2 = 1$, we simulated β such that $\sigma_a^2 = \mathbb{V}\Big(\iota + \sum_{i\in\mathcal{C}} \alpha_0 \mathcal{I}(G_{ij} = 0) + \sum_{i\in\mathcal{C}} \alpha_1 \mathcal{I}(G_{ij} = 1) + \sum_{i\in\mathcal{C}} \alpha_2 \mathcal{I}(G_{ij} = 2)\Big)$, achieving the desired h^2.

B.3 Permutation test

This is done by permuting the observed trait for B times and generate a B-length vector of the TMT statistic. In each round of permutation, let $\{Y_j^*\}$ be the permuted trait values, calculate $d_b = 2\sum_{j=1}^{J}(Y_j^* - \hat{\mu}_c)(W_{1j} - W_{0j})/N$. Then calculate p-value $= \sum_{b=1}^{B} \mathcal{I}(|d_b| \geq |d_{\text{TMT}}|)/B$. Without additional notes, we set the permutation times $B = 1,000$.

B.4 Simulating dichotomous trait

We started from Equation (14) to generate a continuous latent variable $\boldsymbol{L} = \{L_j\}$. Let $\Psi(\cdot)$ be the probit function which is the inverse of standard Normal cumulative distribution function. We standardized L_j as $\tilde{L}_j = \big(L_j - \text{Mean}(\boldsymbol{L})\big)/\text{SD}(\boldsymbol{L})$. Given a disease prevalence λ, $0 < \lambda < 1$, we generated the dichotomous trait value by $Y_j = \mathcal{I}\Big(\Psi\big(\tilde{L}_j > (1 - \lambda)\big)\Big)$.

B.5 Simulating genetic linkage

For the parental genotypes, we used the software `msprime` (version 1.0) [10] and followed the American Admixture model [11] to simulate 5,000 pairs of parents from the admixed Americans popoulation with 100,000 SNPs across 22 chromosomes per individual, each chromosome with 2 haplotypes. Our simulation parameters for `msprime` are the same as parameters listed in the supporting information for [11]. We used the R package `popkin` [1] to calculate the F_{ST} of the parental genotypes, which is around 0.18. We set the total number of SNPs in each chromosome proportional to the corresponding chromosome length in Human Genome Assembly GRCh38.p14. We set the mutation rate per generation so that the allele frequency is between 0.05 and 0.95. We set the recombination rate so that the level of LD matches previous findings in human genome [12, 13]. Within each family, for each chromosome, we randomly drew a haplotype per parental side and merge these two haplotypes as the child genotypes.

B.6 Simulating confounding effects

We followed Appendix B.1 to simulate parental genotypes $\boldsymbol{Z}^m, \boldsymbol{Z}^p$ and child genotypes $\boldsymbol{G}$ with $F_{\mathrm{ST}} = 0.01, 0.05$ for the underlying population. Let $\boldsymbol{Q}$ be the random variable that has confounding effects on the relationship between $\boldsymbol{G}$ and Y. We considered the confounding effects from parental genotypes via $Q_{ij} = Z_{ij}^m + Z_{ij}^p$. Let $\mathcal{C}$ be the set of all causal SNPs and $\mathcal{U}$ the set of non-causal confounding SNPs. We denote the size of these two sets by $C = |\mathcal{C}|$ and $U = |\mathcal{U}|$. We generated the child trait by

$$Y_j = \iota + \sum_{i \in \mathcal{C}} bG_{ij} + \sum_{i \in \mathcal{U}} vQ_{ij} + \epsilon_j,$$

where we drew ϵ_j from Normal$(0, \sigma_e^2)$ and we set $\sigma_e^2 = 1$, $\iota = 100$. The coefficients b and v were determined such that $\mathbb{V}\left(\sum_{i \in \mathcal{C}} bG_{ij}\right) : \mathbb{V}\left(\sum_{i \in \mathcal{U}} vQ_{ij}\right) : \sigma_e^2 = 3 : 3 : 4$.

C Supplementary Figures

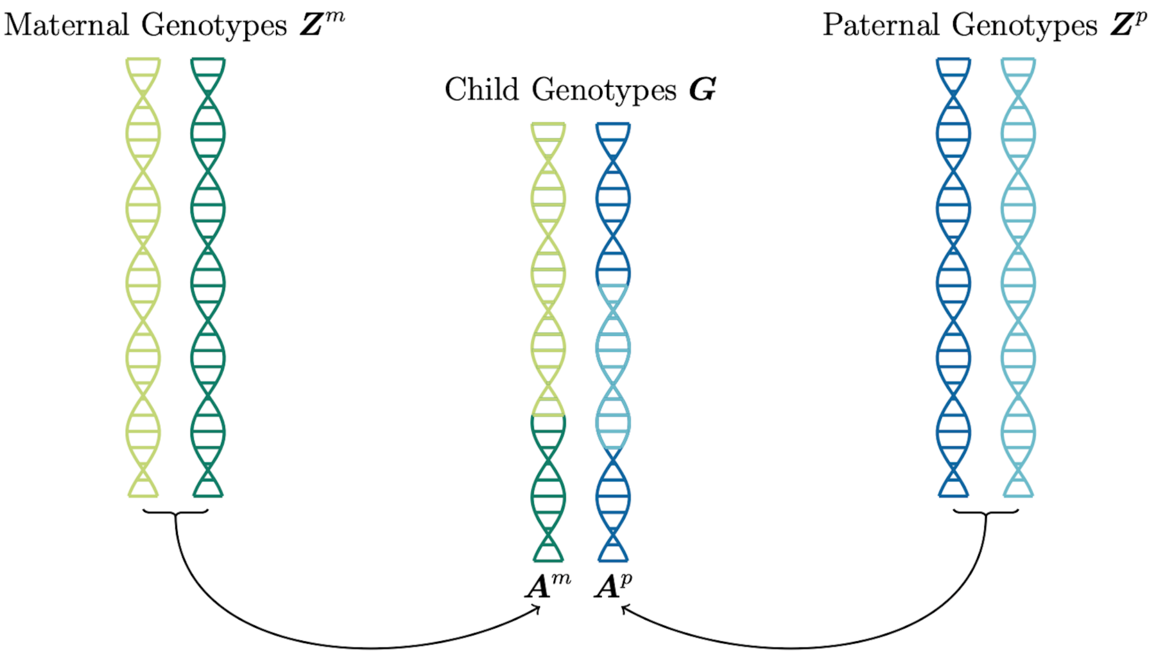

Figure S1: **Schematic of a typical set of trio data and the meiosis process.** The transmission of alleles from heterozygous parents to the child motivates the randomized experiments to identify causal relationship between the child's phenotypes and genotypes. Recombination events may occur, marked as the adjacent dark and light colored pieces for the child's genotypes.

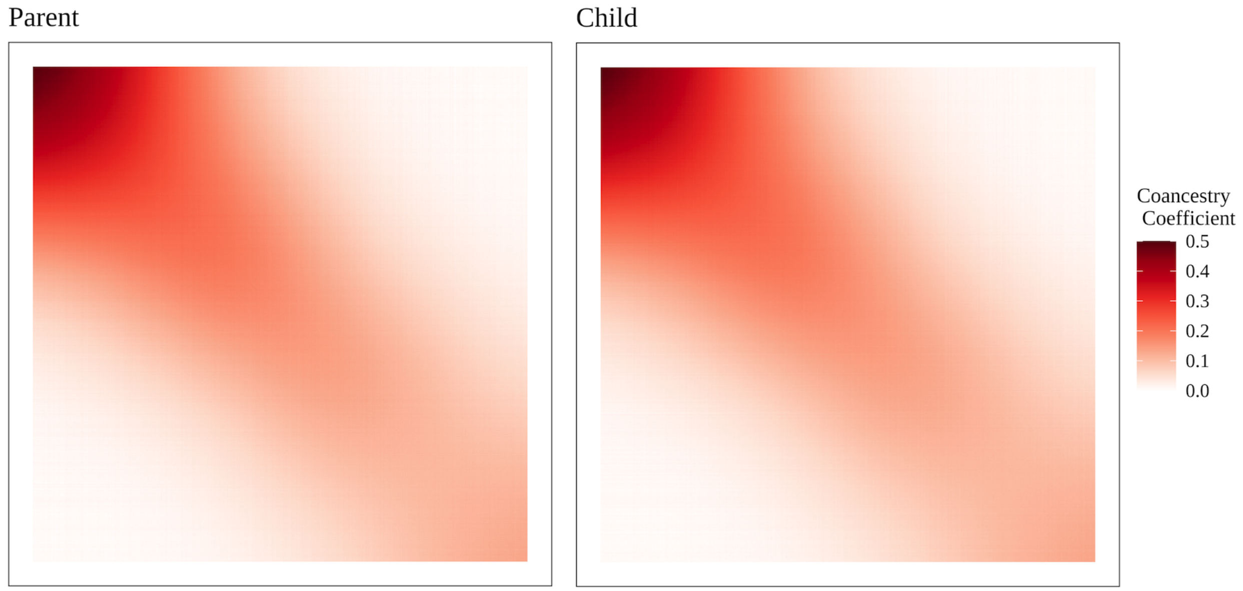

Figure S2: **The co-ancestry coefficients between individuals among the structured population.** Along both x-axis and y-axis lined up the individuals, mothers for the left panel and children for the right panel. Each entry is a pair-wise coancestry coefficient calculated by `popkin` [1] for a simulated sample of 500 trios randomly drawn from the structured population. Here we presented a sample of 500 trios due to the limited figure size. Simulations throughout the paper with larger sample sizes share the same pattern of population structure and the same variability of coancestry coefficients with the overall $F_{ST} = 0.2$.

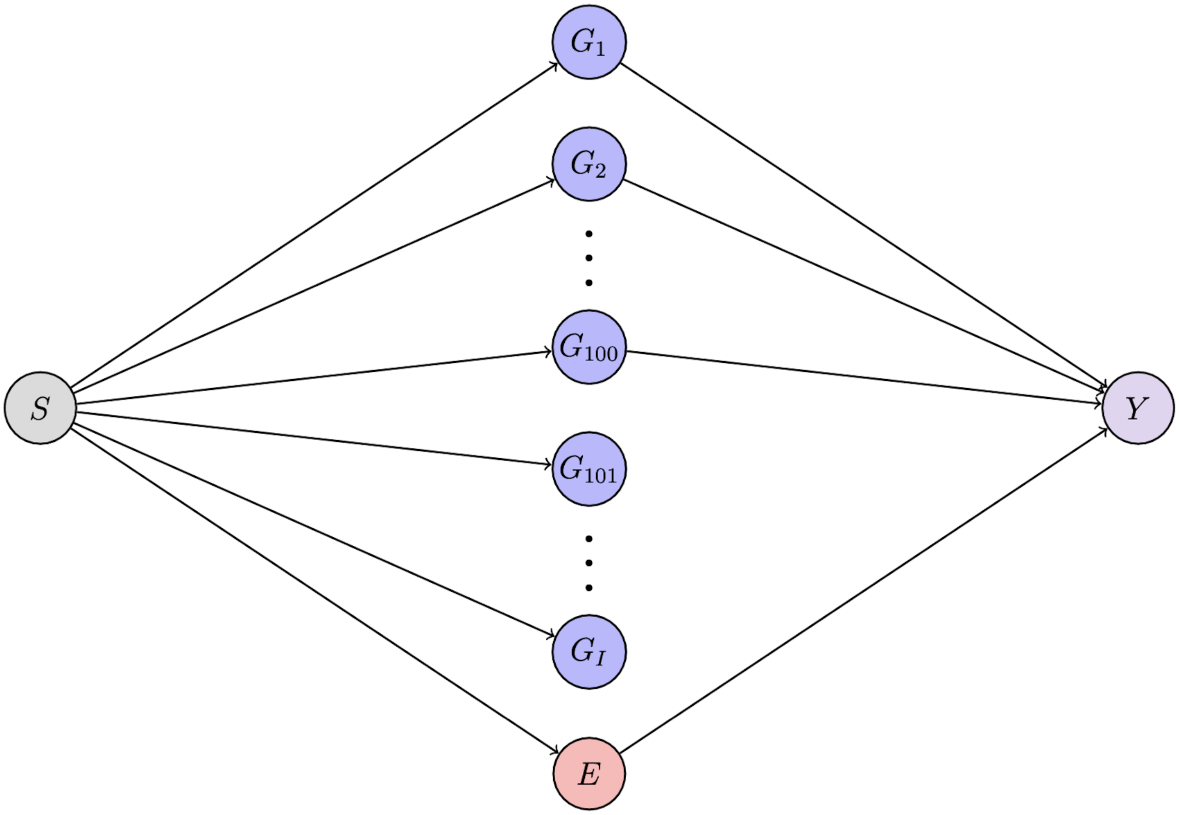

Figure S3: **Schematic of the data simulation model.** The phenotype Y is a function of 100 directly causal loci $(G_1, G_2, \ldots, G_{100})$ and a non-genetic factor E. The remaining loci $(G_{101}, G_{102}, \ldots, G_I)$ are not causal for Y. All non-genetic and genetic variables $(E, G_1, G_2, \ldots, G_I)$ are probabilistically dependent according to the population structure, represented by the variable S.

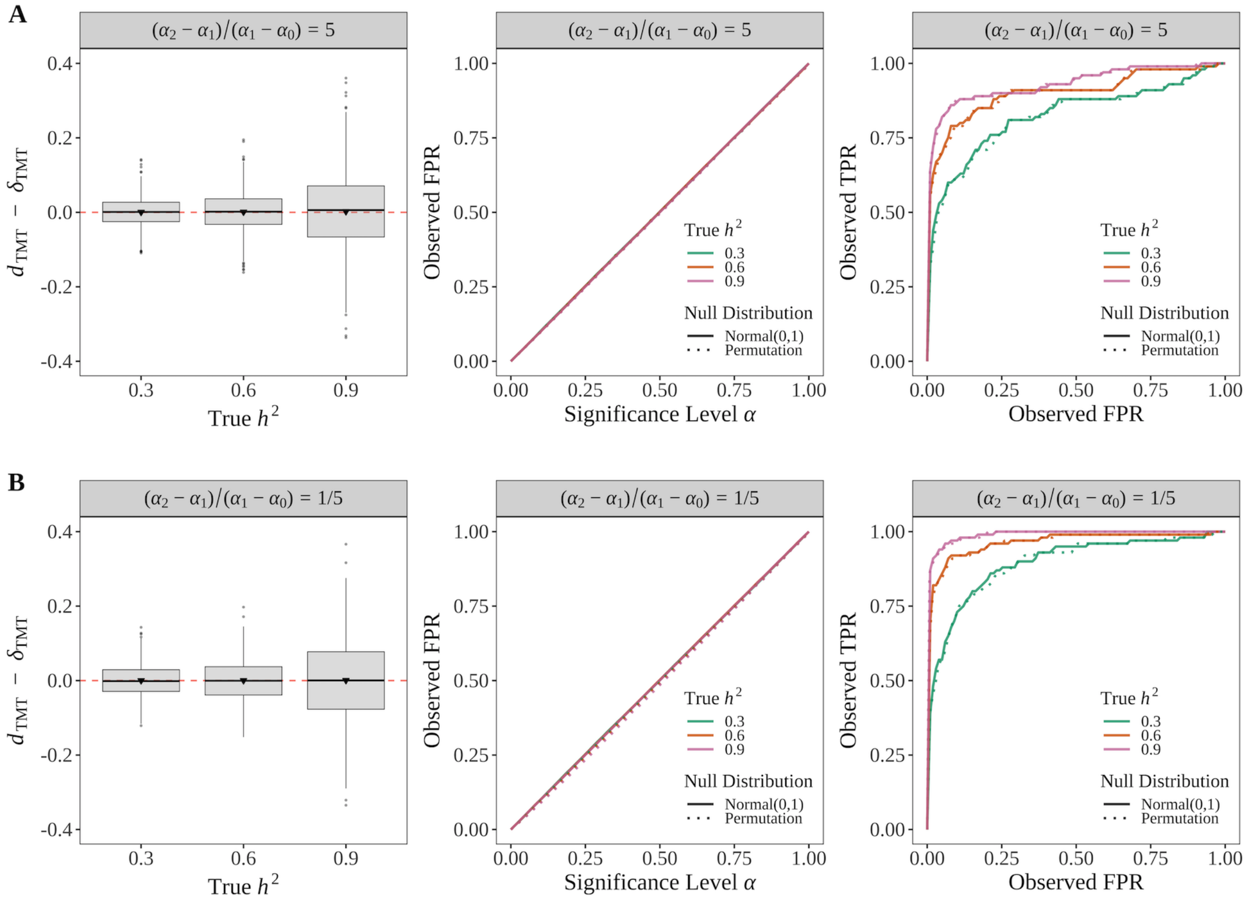

Figure S4: **The TMT delivers unbiased causal effect estimand d_{TMT}, controls FPR at the desired significance level, and has reliable ROC curve across various levels of heritability h^2.** **(A)** Simulation results for $(\alpha_2 - \alpha_1)/(\alpha_1 - \alpha_0) = 5$. **(B)** Simulation results for $(\alpha_2 - \alpha_1)/(\alpha_1 - \alpha_0) = 1/5$. We followed Appendix B.1 to draw $5,000$ trios from an admixed population ($F_{ST} = 0.2$) with $100,000$ SNPs and 100 causal loci per individual. We simulated quantitative traits by following Appendix B.2.

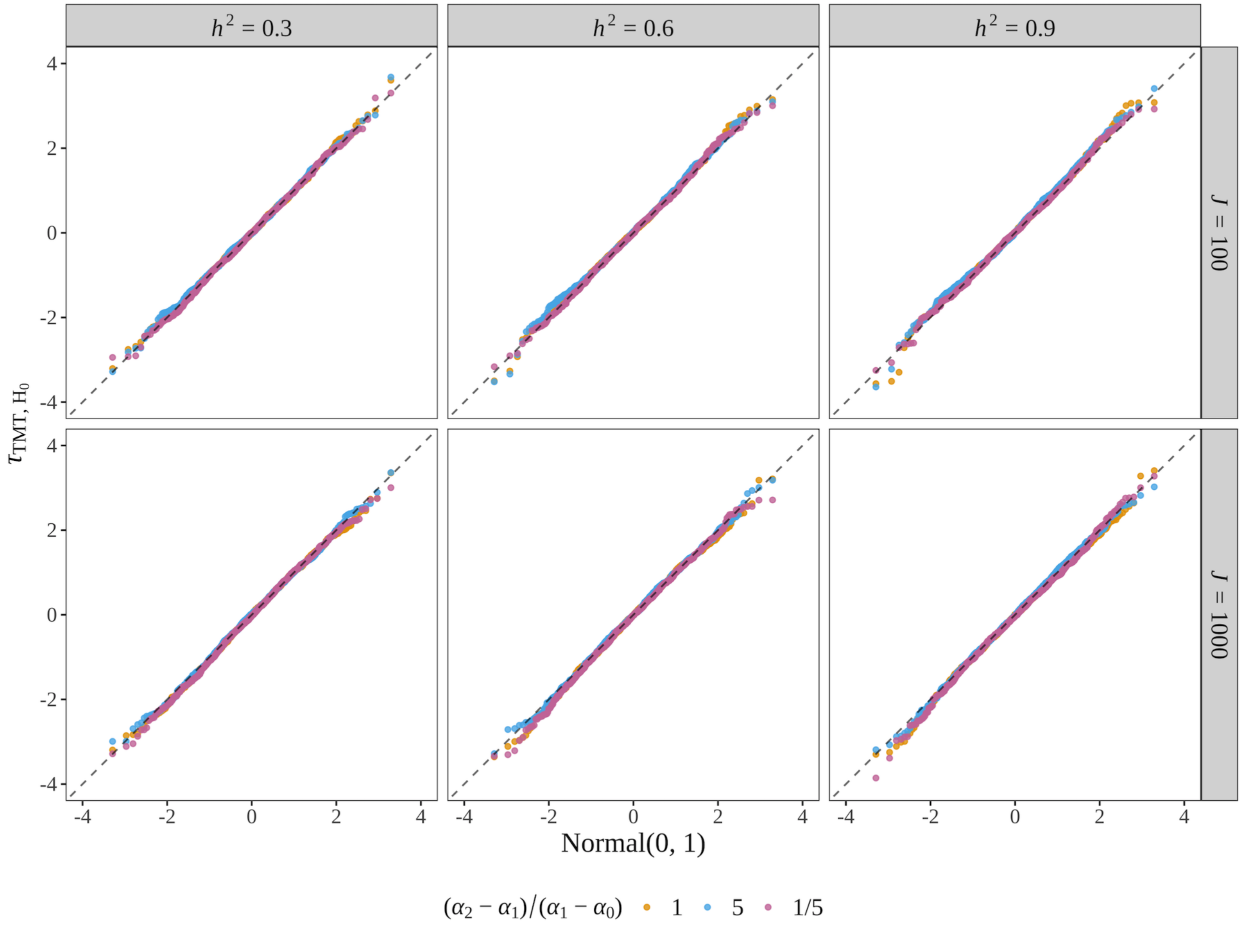

Figure S5: **The Q-Q plot for the statistic τ_{TMT} under null.** We followed Appendix B.1 to draw 100 and $1,000$ trios from an admixed population ($F_{ST} = 0.2$) with $100,000$ SNPs and 100 causal loci per individual. The desired h^2 is 0.5. We simulated quantitative traits by following Appendix B.2. For a randomly chosen causal locus, we conducted 1,000 permutations on trait values and calculated the statistic τ_{TMT} per permutation to generate the observed null.

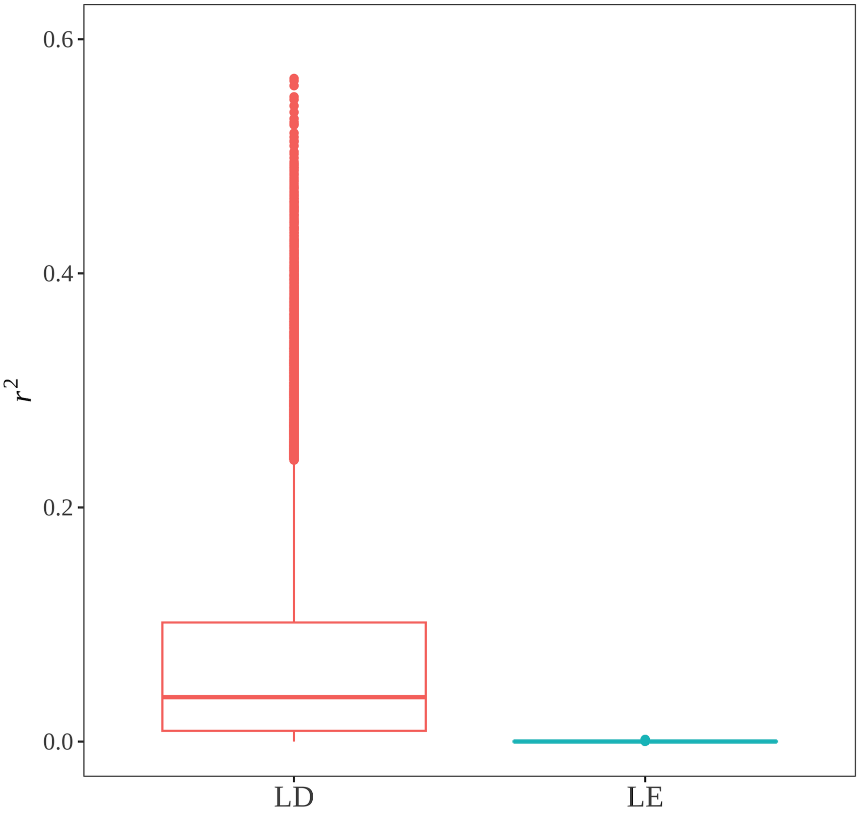

Figure S6: **The distribution of r^2 between adjacent SNPs.** The r^2 values are shown for all pairs of adjacent SNPs for the simulated linkage-disequilibrium (LD) scenario and the linkage-equilibrium (LE) scenario after permutation.

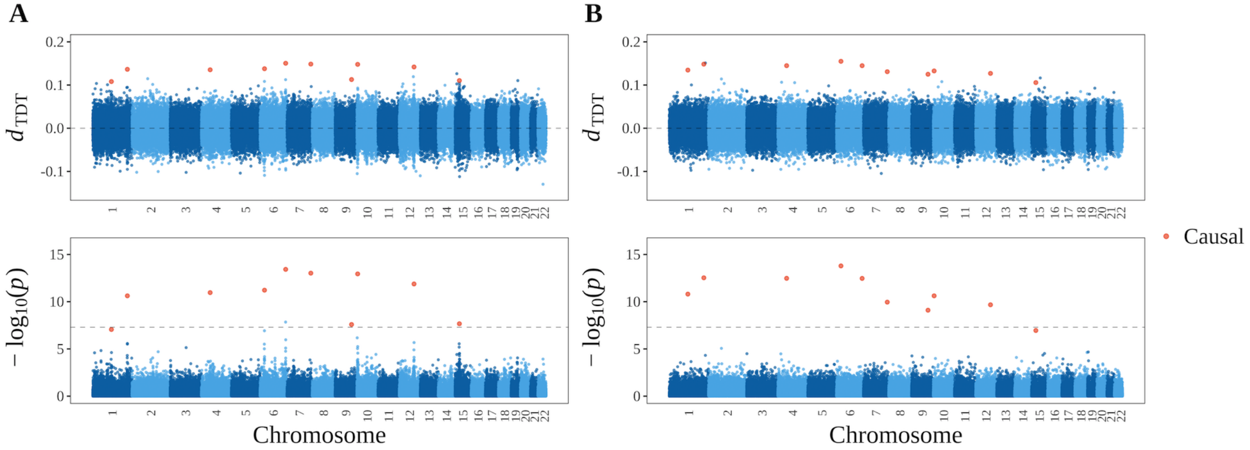

Figure S7: **The genome-wide TDT profile for 2,500 affected-only trios. (A)** Randomization linkage scenario. **(B)** Independent randomization scenario, where the genotypes from (A) were independently permuted to remove the randomization linkage. Genotypes of 5,000 trios are simulated by Appendix B.5. For both scenarios, the top 2,500 children with the highest value of $\iota + \sum_{i \in \mathcal{C}} bG_{ij} + \epsilon_j$ are assigned as affected, setting the trait $Y_j = 1$ and including in the TDT analysis. We draw ϵ_j from Normal$(0, \sigma_e^2)$, set $\iota = 100$, $\sigma_e^2 = 1$ and determine b such that $\mathbb{V}\left(\sum_{i \in \mathcal{C}} bG_{ij}\right) / \sigma_e^2 = 1$. The TDT profile is presented as d_{TDT} and $-\log_{10}(p)$ at 100,000 SNPs across 22 chromosomes simulated by `msprime`. The gray dashed line in the bottom panel is at p-value $= 5 \times 10^{-8}$, which is commonly used as a p-value threshold in GWAS.

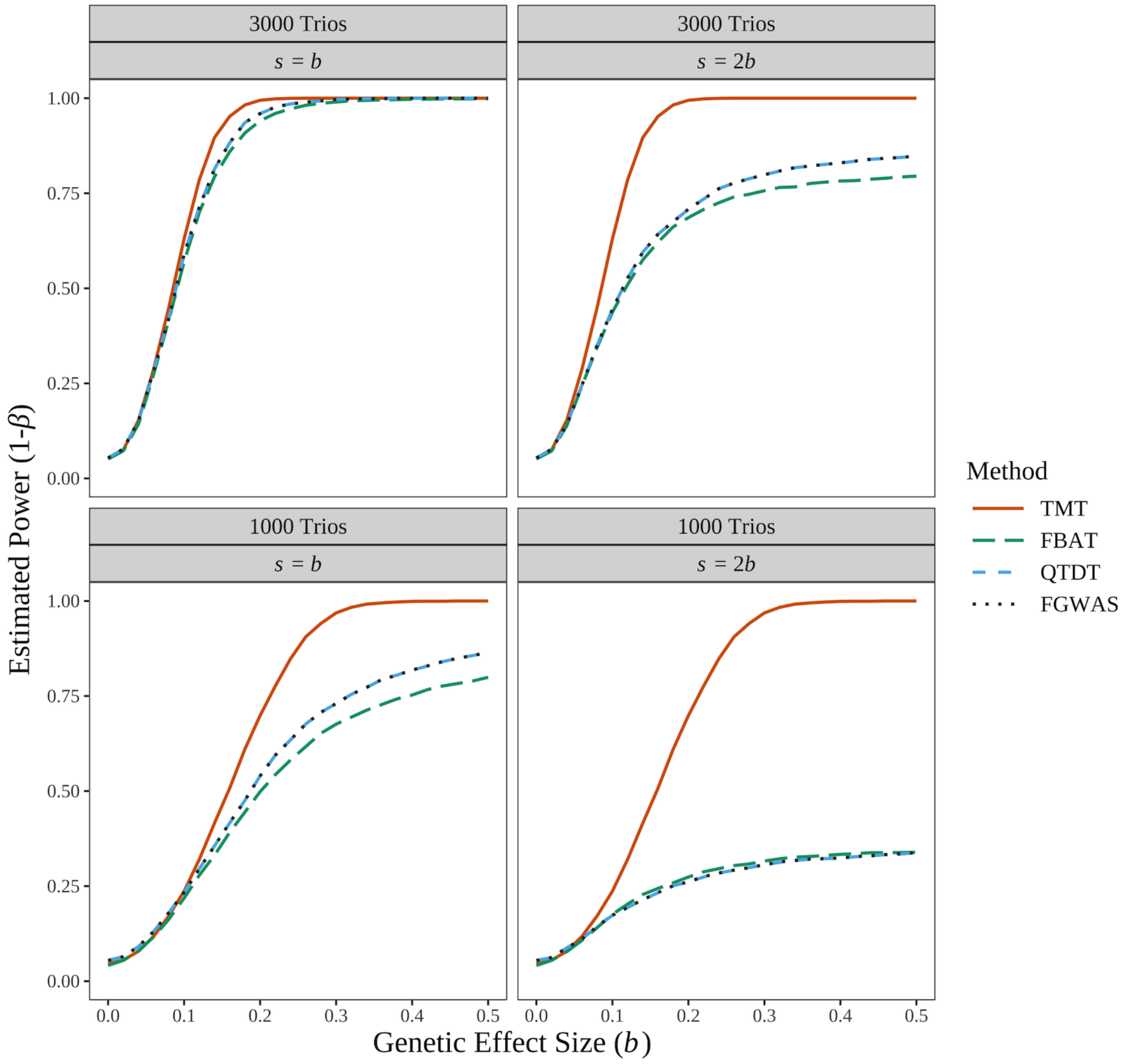

Figure S8: **Statistical power for detecting causal effects of the TMT, FBAT, QTDT and FGWAS in the presence of confounding.** See details in Section 3.4.

References

[1] Alejandro Ochoa and John D Storey. "Estimating FST and kinship for arbitrary population structures". *PLoS Genetics* 17(1) (2021), e1009241.

[2] Augustine Kong et al. "The nature of nurture: Effects of parental genotypes". *Science* 359(6374) (2018), pp. 424–428.

[3] Alexander I Young et al. "Mendelian imputation of parental genotypes improves estimates of direct genetic effects". *Nature Genetics* 54(6) (2022), pp. 897–905.

[4] Carl Veller and Graham M Coop. "Interpreting population-and family-based genome-wide association studies in the presence of confounding". *PLoS Biology* 22(4) (2024), e3002511.

[5] Carl Veller, Molly Przeworski, and Graham Coop. "Causal interpretations of family GWAS in the presence of heterogeneous effects". *Proceedings of the National Academy of Sciences* 121(38) (2024), e2401379121.

[6] Jonathan K Pritchard, Matthew Stephens, and Peter Donnelly. "Inference of population structure using multilocus genotype data". *Genetics* 155(2) (2000), pp. 945–959.

[7] David H Alexander, John Novembre, and Kenneth Lange. "Fast model-based estimation of ancestry in unrelated individuals". *Genome Research* 19(9) (2009), pp. 1655–1664.

[8] Irineo Cabreros and John D Storey. "A likelihood-free estimator of population structure bridging admixture models and principal components analysis". *Genetics* 212(4) (2019), pp. 1009–1029.

[9] Alejandro Ochoa and John D Storey. *Package 'bnpsd'*. R package version 1.3.13. 2021.

[10] Franz Baumdicker et al. "Efficient ancestry and mutation simulation with msprime 1.0". *Genetics* 220(3) (2022), iyab229.

[11] Sharon R Browning et al. "Ancestry-specific recent effective population size in the Americas". *PLoS Genetics* 14(5) (2018), e1007385.

[12] David E Reich et al. "Linkage disequilibrium in the human genome". *Nature* 411(6834) (2001), pp. 199–204.

[13] Elisabeth Dawson et al. "A first-generation linkage disequilibrium map of human chromosome 22". *Nature* 418(6897) (2002), pp. 544–548.
